# Semisynthetic derivatives of massarilactone D with cytotoxic and nematicidal activities

**DOI:** 10.3762/bjoc.21.48

**Published:** 2025-03-17

**Authors:** Rémy B Teponno, Sara R Noumeur, Marc Stadler

**Affiliations:** 1 Department Microbial Drugs, Helmholtz Centre for Infection Research, Inhoffenstraße 7, 38124 Braunschweig, Germany and Institute of Microbiology, Technische Universität Braunschweig, Spielmannstraße 7, 38106 Braunschweig, Germany; 2 Department of Chemistry, Faculty of Science, University of Dschang, P.O. Box 67, Dschang, Cameroon; 3 Laboratoire de biotechnologie des molécules bioactives et de la physiopathologie cellulaire (LBMBPC), Faculté des sciences de la nature et de la vie, Université de Batna 2, Batna 05078, Algeria

**Keywords:** acylation reaction, cytotoxic activity, massarilactone D, nematicidal activity

## Abstract

Massarilactones constitute a rare class of polyketides produced mainly by endophytic fungi. Given that semisynthetic derivatives often exhibit biological activities greater than those of the substrates, seven previously unreported derivatives of massarilactone D, compounds **2**–**8**, were synthetized by acylation with methacryloyl chloride, cinnamoyl chloride, 4-bromobenzoyl chloride, *trans*-2-methyl-2-butenoyl chloride, and crotonyl chloride. These compounds were evaluated for their cytotoxic activity against the murine fibroblasts L929, human cervix carcinoma KB-3-1, human lung carcinoma A549, human prostate cancer PC-3, epidermoid carcinoma A431, ovarian carcinoma SKOV-3, and breast cancer MCF-7 cell lines. Compounds **2** and **3** exhibited significant cytotoxicity against all the tested cells. Some of the semisynthetic derivatives were also tested for their nematicidal activity and compound **4** displayed signiﬁcant and selective nematicidal activity with LD_90_ and LD_50_ of 100 and 12.5 µg/mL, respectively. Since the parent compound was not active, the present study supports the fact that the acylation reaction can improve bioactivities of some natural products.

## Introduction

Cancer continues to be responsible for morbidity and mortality all over the world. Endophytic fungi have been shown to be an important source of secondary metabolites endowed with interesting cytotoxic activities. However, resistance to cancer therapies is a persistent challenge in clinical practice. This resistance often leads to treatment failure and poor survival outcomes for patients [1]. Another ongoing problem is the excessive use of chemical pesticides such as methyl bromide, carbamates, and organophosphates to control plant-parasitic nematodes that has shown a negative impact on the environment and human health. Prolonged and widespread applications of these substances have also increased the development of nematode resistance to pesticides [2–4]. However, advancements in natural products chemistry have shown that chemical modifications of certain natural products can serve as effective scaffold for the design and synthesis of derivatives with improved biological activities [5–7].

**Scheme 1:**
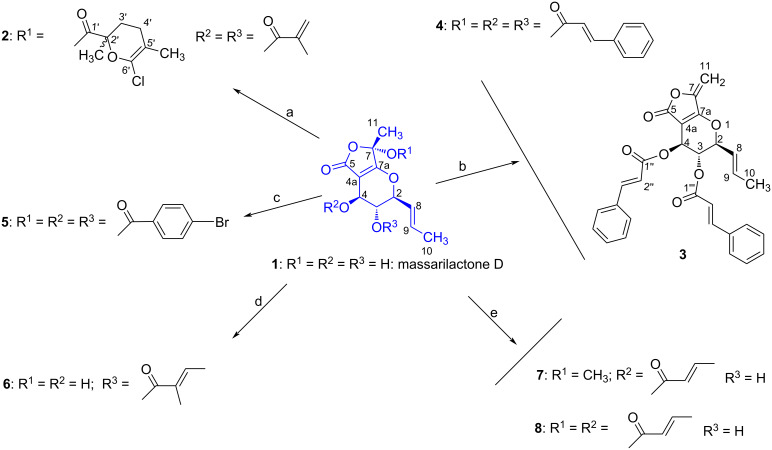
Preparation of massarilactone D derivatives **2**–**8**. Reagents and reactions conditions: a) (CH_3_CH_2_)_3_N/DMAP and methacryloyl chloride in CH_2_Cl_2_; b) (CH_3_CH_2_)_3_N/DMAP and cinnamoyl chloride in CH_2_Cl_2_; c) (CH_3_CH_2_)_3_N/DMAP and 4-bromobenzoyl chloride in CH_2_Cl_2_; d) (CH_3_CH_2_)_3_N/DMAP and *trans*-2-methyl-2-butenoyl chloride in CH_2_Cl_2_; e) (CH_3_CH_2_)_3_N/DMAP and crotonyl chloride in CH_2_Cl_2_.

Massarilactones are produced by marine and endophytic fungi and bear close biogenetic similarity to several other fungal PKS1-derived metabolites including rosigenin, the curvupallides, and the spirostaphylotrichins [8–10]. Massarilactones A and B were isolated for the first time from the freshwater aquatic fungus *Massarina tunicata* [8], massarilactones C and D from *Coniothyrium* sp. associated to the succulent plant *Carpobrotus edulis* [11], massarilactones E, F, and G from *Coniothyrium* sp. associated with the plant *Artimisia maritima* [12] while massarilactone H was first obtained from the marine-derived fungus *Phoma herbarum* [13]. Massarilactones D and H are also produced by the phytopathogenic fungus *Kalmusia variispora* associated with grapevine trunk diseases (GTDs) in Iran [14]. Massarilactones A and B were earlier shown to exhibit antimicrobial activity against *Bacillus subtilis* (ATCC 6051) and *Staphylococcus aureus* (ATCC 29213), affording zones of inhibition varying from 12 to 19 mm at 200 μg/disk [8], while massarilactone H displayed moderate cytotoxicity against three human cancer cell lines, namely A549, Hs683, and SKMEL-28 with IC_50_ of 32.9, 31.5, and 35.2 μM, respectively [15]. Recently, we discovered that massarilactone D was the main secondary metabolite produced during shake flasks fermentation in YMG medium (1.0% malt extract, 0.4% glucose, 0.4% yeast extract, pH 6.3) by the endophytic fungus *Dendrothyrium variisporum*. This fungus, isolated from the roots of the Algerian plant *Globularia alypum,* was explored for the first time for its potential to produce secondary metabolites [16]. Despite the abundant production of massarilactone D, it did not exhibit significant antimicrobial or cytotoxic activities upon testing [16]. This led us to hypothesize that possible chemical modification might enhance its biological activity. Therefore, the present research work aimed to investigate whether structural modifications could improve the biological activity of this polyketide. Seven analogues of massarilactone D, compounds **2**–**8**, were synthetized through acylation modifications aimed at enhancing the compound’s interactions with biological targets (Scheme 1). These acylated analogues were subsequently screened for their cytotoxicity against a panel of human cancer cells and evaluated for their nematicidal activity.

## Results and Discussion

### Semisynthesis of massarilactone D derivatives

The reaction of massarilactone D with methacryloyl chloride in triethylamine in the presence of catalytic amounts of 4-dimethylaminopyridine afforded compound **2** (11% yield) obtained as a white powder. Its molecular formula C_27_H_31_ClO_10_ was deduced from the HRESIMS spectrum (Figure S2, Supporting Information File 1), which exhibited the sodium adduct at *m*/*z* 573.1495 (calcd. for C_27_H_31_ClO_10_Na^+^: 573.1498) and NMR analysis. In addition to resonances attributed to massarilactone D, signals of two methacryloyl moieties were depicted in its ^1^H NMR spectrum at δ_H_ 1.92 (s, Me-2''), 1.89 (s, Me-2'''), 6.24 (dq, *J* = 2.1, 1.0 Hz, Ha-3''), 6.18 (dq, *J* = 2.2, 1.0 Hz, Ha-3'''), 5.80 (p, *J* = 1.6 Hz, Hb-3'''), and 5.73 (p, *J* = 1.6 Hz, Hb-3'') [17]. In the ^13^C NMR spectrum, the presence of these groups was evidenced by resonances observed at δ_C_ 166.4 (C-1''), 164.8 (C-1'''), 136.4 (C-2'', C-2'''), 128.4 (C-3'''), 128.0 (C-3''), 18.3 (Me-2''), and 18.1 (Me-2'''). The HMBC correlations from H-3 (δ_H_ 5.23, t, *J* = 3.4 Hz) and H-4 (δ_H_ 5.75, dd, *J* = 3.3, 1.3 Hz) to carbons at δ_C_ 164.8 (C-1''') and 166.4 (C-1''), respectively, revealed that the two methacryloyl moieties were linked at C-2 and C-3. The other salient feature of the ^13^C NMR spectrum was the presence of some signals at δ_C_ 171.7 (C-1'), 82.3 (C-2'), 31.4 (C-3'), 26.0 (C-4'), 105.1 (C-5'), 137.0 (C-6'), 25.1 (Me-2'), and 18.2 (Me-5') characteristic of a 6-chloro-3,4-dihydro-2,5-dimethyl-2*H*-pyran-2-carbonyl moiety [18]. The chemical shift of C-1' (δ_C_ 171.7) in compound **2** compared to 6-chloro-3,4-dihydro-2,5-dimethyl-2*H*-pyran-2-carbonyl chloride (δ_C_ 176.8) indicated that the 6-chloro-3,4-dihydro-2,5-dimethyl-2*H*-pyran-2-carbonyl group was linked through an ester bond and the only hydroxy group available for this esterification was the one at C-7. This compound was finally elucidated as massarilactone D 3,4-di-*O*-methacryloyl-7-*O*-(6-chloro-3,4-dihydro-2,5-dimethyl-2*H*-pyran-2-carbonyl).

For the formation of compound **2**, an oxa-Diels–Alder reaction between two methacryloyl chloride molecules could have taken place to yield 6-chloro-3,4-dihydro-2,5-dimethyl-2*H*-pyran-2-carbonyl chloride as previously described [18] before esterification of the hydroxy group at C-7.

Compounds **3** (6% yield) and **4** (90% yield) were obtained each as white powder after HPLC purification of the mixture formed from reaction of massarilactone D with cinnamoyl chloride in triethylamine in the presence of catalytic amounts of 4-dimethylaminopyridine. The ESIMS of compound **3** exhibited the sodium adducts at *m*/*z* 507.20 [M + Na]^+^ and 991.39 [2M + Na]^+^ corresponding to the molecular formula C_29_H_24_O_7_. The presence of two cinnamoyl moieties was supported by resonances shown at δ_H_ 7.77 (d, *J* = 16.0 Hz, H-3'''), 7.76 (d, 16.0, H-3''), 7.72–7.70 (o, H-5'', H-9'', H-5''', H-9'''), 7.46–7.44 (o, H-6'', H-7'', H-8'', H-6''', H-7''', H-8'''), 6.60 (d, *J* = 16.0 Hz, H-2'''), and 6.58 (d, *J* = 16.0 Hz, H-2''). In the ^13^C NMR spectrum, the cinnamoyl moieties were evidenced by signals observed at δ_C_ 165.9 (C-1''), 165.7 (C-1'''), 147.4 (C-3''), 147.0 (C-3'''), 135.3 (C-4''), 135.2 (C-4'''), 131.7 (C-7'', C-7'''), 130.0 (C-6'', C-8'', C-6''', C-8'''), 129.4 (C-5'', C-9''), 129.3 (C-5''', C-9'''), 118.0 (C-2''), and 117.8 (C-2''') [19–20]. The HMBC correlations were observed from H-3 (δ_H_ 5.43, t, *J* = 3.6 Hz) to C-1''' (δ_C_ 165.7) and from H-4 (δ_H_ 5.90, o) to C-1'' (δ_C_ 165.9). The ^1^H NMR spectrum of compound **3** revealed the presence of two signals at δ_H_ 5.28 (d, *J* = 3.0 Hz, Ha-11) and 5.23 (d, *J* = 3.0 Hz, Hb-11) characteristic of *exo*-methylene protons as in massarilactone H [13,21], suggesting an *exo*-dehydration of the C-7 hydroxy group. This was further supported by resonances depicted at δ_C_ 148.8 (C-7) and 94.3 (C-11). Since compound **3** is massarilactone H 3,4-di-*O*-*trans*-cinnamoyl, these results suggested that massarilactone H could be an artifact formed during the extraction/isolation of massarilactone D.

The molecular formula of compound **4** was deduced to be C_38_H_32_O_9_ from its HRESIMS spectrum (Figure S19, Supporting Information File 1) which showed the protonated molecular ion at *m*/*z* 633.2077 [M + H]^+^ (calcd. for C_38_H_32_O_9_^+^: 633.2119). This implies that compound **4** has 390 more atomic mass units than massarilactone D, suggesting the presence of three additional cinnamoyl groups. The presence of three cinnamoyl moieties was supported by carbon signals observed at δ_C_ 165.8 (C-1''), 165.3 (C-1'''), 164.6 (C-1'), 148.0 (C-3'), 147.3 (C-3'''), 146.9 (C-3''), 135.3 (C-4'''), 135.2 (C-4''), 134.8 (C-4'), 131.9 (C-7''), 131.6 (C-7', C-7'''), 130.3 (C-6'', C-8''), 130.0 (C-6''', C-8'''), 129.9 (C-6', C-8'), 129.4 (C-5'', C-9'', C-5''', C-9'''), 129.3 (C-5', C-9') and 117.9 (C-2', C-2'', C-2''') [19–20]. Careful examination of ^1^H, ^13^C, ^1^H-^1^H COSY, HSQC, and HMBC spectra revealed compound **4** to be massarilactone D 3,4,7-tri-*O*-*trans*-cinnamoyl.

The molecular formula of compound **5** (product of the reaction of massarilactone D with 4-bromobenzoyl chloride in triethylamine in the presence of catalytic amounts of 4-dimethylaminopyridine, 82% yield) was proposed as C_32_H_23_Br_3_O_9_ based on the molecular ion cluster sodium adducts [M + Na]^+^ depicted at *m*/*z* 810.96, 812.94, 814.92, and 816.90 characteristic for the presence of three bromine atoms [22]. In addition to signals ascribed to the parent compound, the ^1^H NMR spectrum exhibited some resonances at δ_H_ 8.02 (d, *J* = 8.5 Hz, H-3'', H-7''), 7.87 (d, *J* = 8.9 Hz, H-3''', H-7'''), 7.83 (d, *J* = 8.5 Hz, H-3', H-7'), 7.61 (d, *J* = 8.5 Hz, H-4', H-6'), 7.60 (d, *J* = 8.9 Hz, H-4''', H-6'''), and 7.58 (d, *J* = 8.5 Hz, H-4'', H-6'') ascribed to three bromobenzoyl moieties [23]. Furthermore, signals of three carbonyl groups were observed at δ_C_ 164.3 (C-1''), 164.2 (C-1'''), and 162.6 (C-1') in the ^13^C NMR spectrum. On the basis of the above spectroscopic data and by interpretation of the 2D spectra, compound **5** was identified as massarilactone D 3,4,7-tri-*O*-bromobenzoyl.

Compound **6** was obtained in 38% yield from the reaction of massarilactone D and *trans*-2-methyl-2-butenoyl chloride in triethylamine with catalytic amounts of 4-dimethylaminopyridine. Its HRESIMS (Figure S36, Supporting Information File 1) displayed two sodium adducts at *m*/*z* 347.1098 [M + Na]^+^ and 671.2314 [2M + Na]^+^ corresponding to the molecular formula C_16_H_20_O_7_ (calcd. for C_16_H_20_O_7_Na^+^: 347.1101). The NMR data of compound **6** showed the presence of a *trans*-2-methyl-2-butenoyl moiety when compared to those of the parent compound [24]. This was revealed by proton signals observed at δ_H_ 6.92 (dddd, *J* = 8.5, 7.1, 5.6, 1.5 Hz, H-3'), 1.81 (dq, *J* = 7.1, 1.2 Hz, 3H-4'), and 1.77 (q, *J* = 1.2 Hz, 3H-5') as well as carbon resonances depicted at δ_C_ 165.6 (C-1'), 141.0 (C-3'), 128.8 (C-2'), 14.7 (C-4'), and 12.0 (C-5'). Due to the upfield shifts of H-3 (δ_H_ 3.82, dd, *J* = 5.1, 4.4 Hz) and H-4 (δ_H_ 4.38, dd, *J* = 4.3, 0.9 Hz, H-4), the *trans*-2-methyl-2-butenoyl moiety was placed at C-7. The above spectroscopic data combined to the analysis of 2D experiments led to the elucidation of the structure of compound **6** as massarilactone D 7-*O*-*trans*-2-methyl-2-butenoyl.

The reaction of massarilactone D and crotonyl chloride in triethylamine with catalytic amounts of 4-dimethylaminopyridine afforded compounds **7** (16% yield) and **8** (32% yield). Compound **7** was obtained as white powder and its molecular formula C_16_H_20_O_7_ was deduced from its positive-ion mode HRESIMS (Figure S45, Supporting Information File 1) which showed the protonated molecular ion peak and the sodium adduct at *m*/*z* 325.1281 [M + H]^+^ (calcd. for C_16_H_21_O_7_^+^: 325.1282) and 347.1103 (calcd. for C_16_H_20_O_7_Na^+^: 347.1101), respectively. Its ^1^HNMR spectrum displayed in addition to signals ascribed to massarilactone D, those of a crotonyl moiety at δ_H_ 6.97 (dq, *J* = 15.5, 7.1 Hz, H-3''), 5.84 (dq, *J* = 15.5, 1.7 Hz, H-2''), and 1.88 (dd, *J* = 6.9, 1.7 Hz, 3H-4'') and a singlet at δ_H_ 3.20 (s, OMe) attributable to a methoxy group. In the ^13^C NMR spectrum, the presence of these groups was revealed by resonances observed at δ_C_ 165.6 (C-1''), 146.8 (C-3''), 122.9 (C-2''), 51.5 (OMe), and 18.0 (C-4'') [25]. The HMBC correlations observed from H-4 (δ_H_ 5.55, dd, *J* = 3.2, 1.4 Hz) to C-1'' (δ_C_ 165.6) and from the OMe (δ_H_ 3.20, s) to C-7 (δ_C_ 104.4) evidenced the linkage of the methoxy and the crotonyl groups at C-7 and C-4, respectively. Based on the above data, compound **7** was found to be massarilactone D 4-*O*-crotonyl-7-*O*-methyl.

The HRESIMS of the reaction product **8** (Figure S54, Supporting Information File 1) revealed a protonated molecular ion peak at *m*/*z* 379.1383 [M + H]^+^ and a sodium adduct ion peak at *m*/*z* 401.1203 [M + Na]^+^ corresponding to the molecular formula C_19_H_22_O_8_ (calcd. for C_19_H_23_O_8_^+^: 379.1387 and for C_19_H_22_O_8_Na^+^: 401.1207). Apart from resonances attributable to the parent compound, the ^1^H NMR spectrum showed duplicated signals at δ_H_ 7.04 (dq, *J* = 15.5, 6.9, Hz, H-3'), 6.98 (dq, *J* = 15.5, 6.9, Hz, H-3''), 5.85 (dq, *J* = 15.5, 1.7 Hz, H-2', H-2''), 1.90 (dd, *J* = 6.9, 1.7 Hz, 3H-4'), and 1.88 (dd, *J* = 6.9, 1.7 Hz, 3H-4'') ascribed to two crotonyl moieties. In the ^13^C NMR spectrum, the signals depicted at δ_C_ 165.7 (C-1''), 163.9 (C-1'), 149.1 (C-3'), 146.7 (C-3''), 122.9 (C-2''), 122.3 (C-2'), and 18.1 (C-4', C-4'') further confirmed the presence of these groups. The HMBC correlation from H-4 (δ_H_ 5.65, dd, *J* = 4.4, 1.0 Hz) to C-1'' (δ_C_ 165.7) and the upfield shift of H-3 (*δ*_H_ 3.98, dd, *J* = 5.1, 4.4 Hz) compared to the same proton in compounds **2**–**5** evidenced that the crotonyl moieties were linked at C-4 and C-7. Compound **8** was then elucidated as massarilactone D 4,7-di-*O*-crotonyl*.*

### Cytotoxic and nematicidal activities

The antiproliferative properties of massarilactone D and the newly synthesized analogues were evaluated against the murine fibroblasts L929, human cervix carcinoma KB3-1, human lung carcinoma A549, human prostate cancer PC-3, epidermoid carcinoma A431, ovarian carcinoma SKOV-3, and breast cancer MCF-7 cell lines. Although the parent compound was not active as previously described [15–16], compounds **2** and **3** exhibited a significant cytotoxic activity against all the tested cells lines with IC_50_ values ranging from 3.51 to 32.73 μM. Furthermore, compounds **7** and **8** displayed cytotoxicity against the L929 and KB3-1 cell lines with IC_50_ values ranging from 18.50 to 61.73 μM (Table 1). Even if compounds **4**–**6** were not active, the present investigation revealed that the acylation reaction can improve the cytotoxic activity of natural products by increasing the hydrophobicity, enhancing cell membrane permeability and binding affinity with intracellular targets [26]. Structure–activity relationships analysis of both hemisynthetic products **2** and **3** revealed a shared conjugated methylene olefinic function that might be key to their cytotoxic effects. Additionally, compound **3**, which features the *exo*-methylene group at C-7, exhibited greater potency than compound **2**, with the highest cytotoxic activity observed against the MCF-7 cell lines, with IC_50_ values of 3.51 µM for compound **3** compared to 7.09 µM for compound **2**. This suggests that the presence of the *exo*-methylene group is a critical structural feature that influences the cytotoxic activity, as outlined earlier [15]. These data also demonstrate possible selective effects of acylated massarilactone D in biological systems.

**Table 1:** Cytotoxic effect (IC_50_) of compounds **1**–**8** against some cell lines.

Samples	IC_50_ (µM)^a^						

	L929	KB3.1	A549	PC-3	A431	SKOV-3	MCF-7

**1**	na	na	na	na	na	na	na
**2**	5.09	13.09	32.73	11.82	4.00	11.82	7.09
**3**	5.58	7.85	19.63	7.64	4.34	13.22	3.51
**4**	na	na	na	na	na	na	na
**5**	na	na	na	na	na	na	na
**6**	na	na	na	na	na	na	na
**7**	61.73	55.56	na	na	na	na	na
**8**	50.26	18.52	na	na	na	na	na
epothilone B	2.2 × 10^−3^	7.5 × 10^‒5^	5.3 × 10^‒5^	9.1 × 10^−5^	5.9 × 10^‒5^	9.1 × 10^‒5^	7.5 × 10^−5^

^a^na: not active.

Massarilactone D 3,4,7-tri-*O*-bromobenzoyl (**4**) also showed signiﬁcant and selective nematicidal activity with LD_90_ and LD_50_ of 100 and 12.5 µg/mL, respectively. In the case of compound **5**, the LD_90_ was not obtained but the LD_50_ was determined to be 100 µg/mL. Compounds **1**, **6**, **7**, and **8** showed some mortality at concentrations of 50 and 100 μg/mL, but their LD_50_ values were not obtained (Table 2). Interestingly, the derivative massarilactone H 3,4-di-*O*-*trans*-cinnamoyl (**3**), which contains only two cinnamoyl groups, did not exhibit nematicidal activity. In contrast, massarilactone D 3,4,7-tri-*O*-*trans*-cinnamoyl (**4**), featuring three cinnamoyl groups, including an additional substitution on C-7, showed potent activity against nematodes. These acyl substituents, particularly the third cinnamoyl group on C-7, may significantly enhance the biological activity. This modification likely represents a key structural feature influencing its activity, as indicated by structure–activity relationship analysis.

**Table 2:** Nematicidal activity of compounds **1**, and **4**–**8**.

Compounds	LD_90_ (µg/mL)^a^	LD_50_ (µg/mL)^a^

**1**	no	no
**4**	100	12.5
**5**	no	100
**6**	no	no
**7**	no	no
**8**	no	no

^a^no: not obtained.

## Conclusion

In the present study, the use of various acylating reagents to modify massarilactone D introduces distinct functional groups, each with unique chemical properties. This approach led to the synthesis of seven previously undescribed derivatives **2**–**8**. The preliminary characterization of these products, in contrast to the parent compound, showed that both massarilactone D 3,4-di-*O*-methacryloyl-7-*O*-(6-chloro-3,4-dihydro-2,5-dimethyl-2*H*-pyran-2-carbonyl) (**2**) and massarilactone H 3,4-di-*O*-*trans*-cinnamoyl (**3**) exhibited a significant cytotoxic activity against all tested cell lines with IC_50_ values ranging from 3.51 to 32.73 μM. Furthermore, massarilactone D 3,4,7-tri-*O*-*trans*-cinnamoyl (**4**) also displayed a good nematicidal activity against *Caenorhabditis elegans*. This work once again confirms that some hemisynthetic derivatives can be more active than their natural substrates, thus expanding their therapeutic potential for cancer and parasitic infections. However, the effect of sequence variation on activity remains unclear. Additionally, the presence of the massarilactone H core in compound **3** formed from the reaction of massarilactone D with cinnamoyl chloride suggests that these two polyketides can interconvert from each other and could be artifacts and vice versa formed during extraction and isolation. This finding suggests that, to improve the antiproliferative efficacy of these derivatives, the *exo*-methylene group should be preserved, with chemical modifications focusing on massarilactone H rather than massarilactone D. These modifications should target other regions of the molecule, such as the number and position of acyl substituents. Future research should investigate the stability and selectivity of these compounds, as well as detailed structure–activity relationships, to optimize massarilactone hemisynthetic derivatives for specific biological applications.

## Experimental

### General experimental procedures

UV–vis spectra were recorded on a Shimadzu UV/Vis 2450 spectrophotometer. Optical rotations were obtained from an Anton Paar MCP-150 Polarimeter with sodium D line at 589 nm and 100 mm path length. HRESIMS mass spectra were measured with a maXis ESI TOF mass spectrometer (Bruker Daltonics) [scan range *m*/*z* 100–2500, rate 2 Hz, capillary voltage 4500 V, dry temperature 200 °C], coupled to an Agilent 1200 series HPLC-UV system [column 2.1 × 50 mm, 1.7 μm, C18 Acquity UPLC BEH (Waters), solvent A: H_2_O + 0.1% formic acid; solvent B: ACN + 0.1% formic acid, gradient: 5% B for 0.5 min, increasing to 100% B in 19.5 min, maintaining 100% B for 5 min, RF = 0.6 mL/min, UV–vis detection 200–600 nm]. NMR spectra were recorded in deuterated solvents (CDCl_3_ and acetone-*d*_6_) with an Avance III 700 (Bruker, Billerica, MA, USA) (^1^H: 700 MHz, ^13^C: 175 MHz) and an Avance III 500 (Bruker, Bremen, Germany) (^1^H: 500 MHz, ^13^C: 125 MHz) spectrometers. Chemical shifts are given in parts per million (ppm), and coupling constants in hertz (Hz). HPLC-DAD-MS analysis was performed using an amaZon speed ETD ion trap mass spectrometer (Bruker Daltonics) in positive and negative ionization modes. The mass spectrometer was coupled to a DIONEX UltiMate 3000 diode array detector [column 2.1 × 50 mm, 1.7 μm, C18 Acquity UPLC BEH (Waters), solvent A: H_2_O + 0.1% formic acid; solvent B: acetonitrile (ACN) + 0.1% formic acid, gradient: 5% B for 0.5 min, increasing to 100% B in 20 min, maintaining isocratic conditions at 100% B for 10 min, flow = 0.6 mL/min, UV–vis detection 190–600 nm]. Preparative HPLC was achieved at room temperature on an Agilent 1100 series preparative HPLC system [ChemStation software (Rev. B.04.03 SP1); binary pump system; column: Kinetex 5u RP C18, dimensions 250 × 21.20 mm; mobile phase: ACN + 0.05% trifluoroacetic acid (TFA) and water + 0.05% TFA; flow rate: 20 mL/min; diode array UV detector; 226 fraction collector].

### Semisynthesis of massarilactone D derivatives

#### Preparation of compound **2**

Massarilactone D (**1**, 15 mg, 0.062 mmol) was dissolved in CH_2_Cl_2_ (10 mL). Triethylamine (20 μL) and methacryloyl chloride (30 μL, 0.31 mmol) were added to the solution. After adding a catalytic amount of 4-dimethylaminopyridine, the reaction mixture was stirred at room temperature overnight. The reaction mixture was suspended in water (25 mL) and extracted with CH_2_Cl_2_ (2 × 25 mL). The combined organic layer was evaporated to dryness to give a residue, which was dissolved in methanol (300 μL) and purified by preparative HPLC [ChemStation software (Rev. B.04.03 SP1); binary pump system; column: Kinetex 5u RP C18, dimensions 250 × 21.20 mm; mobile phase: ACN + 0.05% trifluoroacetic acid (TFA) and water + 0.05% TFA; flow rate 20 mL/min; diode array UV detector; 226 fraction collector. A gradient from 47 to 72% solvent B in 50 min was used]. Compound **2** was obtained in 11% yield (3.50 mg, *t*_R_ = 39.28 min). White powder; [α]_D_^25^ − 38.8 (*c* 0.0006, acetone); UV (*c* 0.075 mg/mL, EtOH) λ_max_ 241 nm (3.84); ^1^H NMR (500 MHz, acetone-*d*_6_) δ_H_ 6.24 (dq, *J* = 2.1, 1.0 Hz, Ha-3''), 6.18 (dq, *J* = 2.2, 1.0 Hz, Ha-3'''), 6.12–6.08 (m, H-9), 5.93 (ddq, *J* = 15.2, 8.7, 1.6 Hz, H-8), 5.80 (p, *J* = 1.6 Hz, Hb-3'''), 5.75 (dd, *J* = 3.3, 1.3 Hz, H-4), 5.73 (p, *J* = 1.6 Hz, Hb-3''), 5.27 (dd, *J* = 8.7, 3.4 Hz, H-2), 5.23 (t, *J* = 3.4 Hz, H-3), 2.31 (ddd, *J* = 13.7, 6.6, 1.9 Hz, Ha-3'), 2.07 (o, Ha-4'), 2.00 (o, Hb-4'), 1.92 (s, Me-2''), 1.89 (s, Me-2'''), 1.83 (s, 3H-11), 1.83–182 (m, Hb-3'),1.80–178 (m, 3H-10), 1.61 (s, Me-5'), 1.50 (s, Me-2'); ^13^C NMR (175 MHz, acetone-*d*_6_) δ_C_ 175.8 (C-7a), 171.7 (C-1'), 166.4 (C-1''), 166.0 (C-5), 164.8 (C-1'''), 137.0 (C-6'), 136.4 (C-2'', C-2'''), 135.2 (C-9), 128.4 (C-3'''), 128.0 (C-3''), 124.1 (C-8), 105.1 (C-5'), 100.8 (C-7), 99.2 (C-4a), 83.7 (C-2), 82.3 (C-2'), 69.9 (C-3), 62.3 (C-4), 31.4 (C-3'), 26.0 (C-4'), 25.1 (Me-2'), 23.5 (C-11), 18.3 (Me-2''), 18.2 (Me-5'), 18.1 (C-10, Me-2'''); HRESIMS (*m*/*z*): [M + Na]^+^ calcd for C_27_H_31_O_10_NaCl^+^, 573.1498; found, 573.1495.

#### Preparation of compounds **3** and **4**

Massarilactone D (**1**, 15 mg, 0.062 mmol) was dissolved in CH_2_Cl_2_ (10 mL). Triethylamine (20 μL) and cinnamoyl chloride (50 mg, 0.30 mmol) were added to the solution. After adding a catalytic amount of 4-dimethylaminopyridine, the reaction mixture was stirred at room temperature overnight. The reaction mixture was suspended in water (25 mL) and extracted with CH_2_Cl_2_ (2 × 25 mL). The combined organic layer was evaporated to dryness to give a residue, which was dissolved in methanol (300 μL) and purified by preparative HPLC (gradient from 60 to 85% solvent B in 50 min) to yield compounds **3** in 6% yield (1.72 mg, *t*_R_ = 25.24 min) and **4** in 90% yield (35.30 mg, *t*_R_ = 36.34 min).

**Compound 3:** White powder; [α]_D_^25^ +189.6 (*c* 0.00058, acetone); UV (*c* 0.01875 mg/mL, EtOH) λ_max_ 232 nm (4.27), 284 nm (4.59); ^1^H NMR (700 MHz, acetone-*d*_6_) δ_H_ 7.77 (d, *J* = 16.0 Hz, H-3'''), 7.76 (d, *J* = 16.0 Hz, H-3''), 7.72–7.70 (o, H-5'', H-9'', H-5''', H-9'''), 7.46–7.44 (o, H-6'', H-7'', H-8'', H-6''', H-7''', H-8'''), 6.60 (d, *J* = 16.0 Hz, H-2'''), 6.58 (d, *J* = 16.0 Hz, H-2''), 6.07 (dqd, *J* = 15.3, 6.5, 1.1 Hz, H-9), 5.90 (o, H-4, H-8), 5.43 (t, *J* = 3.6 Hz, H-3), 5.28 (d, *J* = 3.0 Hz, Ha-11), 5.23 (d, *J* = 3.0 Hz, Hb-11), 1.78 (ddd, *J* = 6.6, 1.6, 0.6 Hz, 3H-10); ^13^C NMR (125 MH2, acetone-*d*_6_) δ_C_ 165.9 (C-5, C-1''), 165.7 (C-7a, C-1'''), 148.8 (C-7), 147.4 (C-3''), 147.0 (C-3'''), 135.3 (C-4''), 135.2 (C-4'''), 134.1 (C-9), 131.7 (C-7'', C-7'''), 130.0 (C-6'', C-8'', C-6''', C-8'''), 129.4 (C-5'', C-9''), 129.3 (C-5''', C-9'''), 124.6 (C-8), 118.0 (C-2''), 117.8 (C-2'''), 100.1 (C-4a), 94.3 (C-11), 82.9 (C-2), 70.0 (C-3), 62.1 (C-4), 18.1 (C-10); ESIMS *m*/*z*: [M + Na]^+^ 507.20, [2M + Na]^+^ 991.39

**Compound 4:** White powder, [α]_D_^25^ +252.2 (*c* 0.0029, acetone); UV (*c* 0.009375 mg/mL, EtOH) λ_max_ 234 nm (4.69), 284 nm (5.15); ^1^H NMR (700 MHz, acetone-*d*_6_) δ_H_ 7.90 (d, *J* = 16.1 Hz, H-3'''), 7.76 (d, *J* = 16.0 Hz, H-3''), 7.76 (d, *J* = 16.0, H-3'), 7.73–7.69 (o, H-5', H-9', H-5''', H-9'''), 7.57–7.56 (m, H-5'', H-9''), 7.47–7.41 (o, H-6', H-7', H-8', H-6'', H-7'', H-8'', H-6''', H-7''', H-8'''), 6.58 (d, *J* = 16.0 Hz, H-2''), 6.57 (d, *J* = 16.0 Hz, H-2'), 6.55 (d, *J* = 16.1 Hz, H-2'''), 6.09 (dqd, *J* = 15.3, 6.5, 1.1 Hz, H-9), 5.94 (dqd, *J* = 15.3, 8.0, 1.6 Hz, H-8), 5.89 (dd, *J* = 3.2, 1.4 Hz, H-4), 5.43 (t, *J* = 3.3 Hz, H-3), 5.36 (ddt, *J* = 6.9, 3.3, 1.2 Hz, H-2), 1.79 (ddd, *J* = 6.5, 1.7, 0.8 Hz, 3H-10),1.91 (s, 3H-11); ^13^C NMR (125 MHz, acetone-*d*_6_) δ_C_ 175.4 (C-7a), 167.0 (C-5), 165.8 (C-1''), 165.3 (C-1'''), 164.6 (C-1'), 148.0 (C-3'), 147.3 (C-3'''), 146.9 (C-3''), 135.3 (C-4'''), 135.2 (C-4''), 134.8 (C-4'), 134.2 (C-9), 131.9 (C-7''), 131.6 (C-7', C-7'''), 130.3 (C-6'', C-8''), 130.0 (C-6''', C-8'''), 129.9 (C-6', C-8'), 129.4 (C-5'', C-9'', C-5''', C-9'''), 129.3 (C-5', C-9'), 124.2 (C-8), 117.9 (C-2', C-2'', C-2'''), 100.9 (C-7), 99.3 (C-4a), 83.2 (C-2), 69.7 (C-3), 62.2 (C-4), 23.6 (C-11), 18.2 (C-10); HRESIMS (*m*/*z*): [M + H]^+^ calcd for C_38_H_32_O_9_^+^, 633.2119; found, 633.2077.

#### Preparation of compound **5**

Massarilactone D (**1**, 15 mg, 0.062 mmol) was dissolved in CH_2_Cl_2_ (10 mL). Triethylamine (20 μL) and 4-bromobenzoyl chloride (70 mg, 0.32 mmol) were added to the solution. After adding a catalytic amount of 4-dimethylaminopyridine, the reaction mixture was stirred at room temperature overnight. The reaction mixture was suspended in water (25 mL) and extracted with CH_2_Cl_2_ (2 × 25 mL). The combined organic layer was evaporated to dryness to give a residue, which was dissolved in methanol (300 μL) and purified by preparative HPLC (gradient from 60 to 100% solvent B in 50 min) to yield compound **5** in 82% yield (39.79 mg, *t*_R_ = 22.50 min). White powder; [α]_D_^25^ +38.79 (*c* 0.0099, acetone); UV (*c* 0.0375 mg/mL, EtOH) λ_max_ 252 nm (4.80); ^1^H NMR (500 MHz, CDCl_3_) δ_H_ 8.02 (d, *J* = 8.5 Hz, H-3'', H-7''), 7.87 (d, *J* = 8.9 Hz, H-3''', H-7'''), 7.83 (d, *J* = 8.5 Hz, H-3', H-7'), 7.61 (d, *J* = 8.5 Hz, H-4', H-6'), 7.60 (d, *J* = 8.9 Hz, H-4''', H-6'''), 7.58 (d, *J* = 8.5 Hz, H-4'', H-6''), 6.07 (dd, *J* = 2.8, 1.4 Hz, H-4), 5.98 (dqd, *J* = 14.2, 6.6, 1.1 Hz, H-9), 5.73 (dqd, *J* = 15.3, 7.8, 1.5 Hz, H-8), 5.56 (t, *J* = 2.9 Hz, H-3), 5.25 (ddt, *J* = 7.8, 2.9, 1.2 Hz, H-2), 1.74 (ddd, *J* = 6.6, 1.6, 0.9 Hz, 3H-10), 1.99 (s, 3H-11); ^13^C NMR (125 MHz, CDCl_3_) δ_C_ 174.8 (C-7a), 166.2 (C-5), 164.3 (C-1''), 164.2 (C-1'''), 162.6 (C-1'), 133.6 (C-9), 132.0 (C-4', C-6'), 131.8 (C-3'', C-4'', C-6'', C-7'', C-4''', C-6'''), 131.3 (C-3', C-7', C-3''', C-7'''), 129.3 (C-5'), 129.0 (C-5''), 128.8 (C-5'''), 128.0 (C-2'''), 127.6 (C-2', C-2''), 122.4 (C-8), 100.2 (C-7), 98.2 (C-4a), 82.1 (C-2), 69.2 (C-3), 61.8 (C-4), 23.2 (C-11), 18.0 (C-10). ESIMS *m*/*z*: [M + Na]^+^ 810.96

#### Preparation of compound **6**

Massarilactone D (**1**, 15 mg, 0.062 mmol) was dissolved in CH_2_Cl_2_ (10 mL). Triethylamine (20 μL) and *trans*-2-methyl-2-butenoyl chloride (34 μL, 0.30 mmol) were added to the solution. After adding a catalytic amount of 4-dimethylaminopyridine, the reaction mixture was stirred at room temperature overnight. The reaction mixture was suspended in water (25 mL) and extracted with CH_2_Cl_2_ (2 × 25 mL). The combined organic layer was evaporated to dryness to give a residue, which was dissolved in methanol (300 μL) and purified by preparative HPLC (gradient from 20 to 54% solvent B in 50 min) to yield compound **6** in 38% yield (7.52 mg, *t*_R_ = 25.92 min). White powder; [α]_D_^25^ −98.37 (*c* 0.00123, acetone); UV (*c* 0.15 mg/mL, EtOH) λ_max_ 247 nm (3.84); ^1^H NMR (500 MHz, acetone-*d*_6_) δ_H_ 6.92 (dddd, *J* = 8.5, 7.1, 5.6, 1.5 Hz, H-3'), 5.92 (m, H-9), 5.85 (ddq, *J* = 15.3, 8.1, 1.3 Hz, H-8), 4.81 (dd, *J* = 7.6, 5.2 Hz, H-2), 4.38 (dd, *J* = 4.3, 0.9 Hz, H-4), 3.82 (dd, *J* = 5.1, 4.4 Hz, H-3), 1.81 (dq, *J* = 7.1, 1.2 Hz, 3H-4'), 1.77 (q, *J* = 1.2 Hz, 3H-5'), 1.76 (s, 3H-11), 1.72 (m, 3H-10); ^13^C NMR (125 MHz, acetone-*d*_6_) δ_C_ 173.8 (C-7a), 168.0 (C-5), 165.6 (C-1'), 141.0 (C-3'), 132.8 (C-9), 128.8 (C-2'), 126.6 (C-8), 100.4 (C-7), 102.9 (C-4a), 86.3 (C-2), 72.8 (C-3), 64.7 (C-4), 23.3 (C-11), 18.1 (C-10), 14.7 (C-4'), 12.0 (C-5'); HRESIMS (*m*/*z*): [M + Na]^+^ calcd for C_16_H_20_O_7_Na^+^, 347.1101; found, 347.1098

#### Preparation of compounds **7** and **8**

Massarilactone D (**1**, 15 mg, 0.062 mmol) was dissolved in CH_2_Cl_2_ (10 mL). Triethylamine (20 μL) and crotonyl chloride (25 μL, 0.26 mmol) were added to the solution. After adding a catalytic amount of 4-dimethylaminopyridine, the reaction mixture was stirred at room temperature overnight. The reaction mixture was suspended in water (25 mL) and extracted with CH_2_Cl_2_ (2 × 25 mL). The combined organic layers were evaporated to dryness to give a residue, which was dissolved in methanol (300 μL) and purified by preparative HPLC (gradient from 26 to 50% solvent B in 50 min) to yield compounds **7** in 16% yield (3.2 mg, *t*_R_ = 31.50 min) and **8** in 32% yield (7.3 mg, *t*_R_ = 46.23 min).

**Compound 7:** White powder; [α]_D_^25^ +25.97 (*c* 0.00077, acetone); UV (*c* 0.01875 mg/mL, EtOH) λ_max_ 241 nm (4.41); ^1^H NMR (500 MHz, acetone-*d*_6_) δ 6.97 (dq, *J* = 15.5, 7.1 Hz, H-3''), 5.92 (m, H-9), 5.84 (dq, *J* = 15.5, 1.7 Hz, H-2''), 5.73 (ddq, *J* = 15.5, 7.7, 1.6 Hz, H-8), 5.55 (dd, *J* = 3.2, 1.4 Hz, H-4), 5.12 (ddt, *J* = 6.6, 3.3, 1.2 Hz, H-2), 4.06 (t, *J* = 3.2 Hz, H-3), 3.20 (s, OMe), 1.88 (dd, *J* = 6.9, 1.7 Hz, 3H-4''), 1.73 (ddd, *J* = 6.5, 1.6, 0.9 Hz, 3H-10), 1.63 (s, 3H-11); ^13^C NMR (125 MHz, acetone-*d*_6_) δ_C_ 174.1 (C-7a), 168.1 (C-5), 165.6 (C-1''), 146.8 (C-3''), 132.0 (C-9), 125.7 (C-8), 122.9 (C-2''), 104.4 (C-7), 100.3 (C-4a), 85.3 (C-2), 69.1 (C-3), 63.5 (C-4), 51.5 (OMe), 22.8 (C-11), 18.1 (C-10), 18.0 (C-4''); HRESIMS (*m*/*z*): [M + H]^+^ calcd for C_16_H_21_O_7_^+^, 325.1282; found, 325.1281; [M + Na]^+^ calcd for C_16_H_20_O_7_Na^+^, 347.1101; found, 347.1103.

**Compound 8:** White powder; [α]_D_^25^ −34.74 (*c* 0.0019, acetone); UV (*c* 0.075 mg/mL, EtOH) λ_max_ 241 nm (4.00); ^1^H NMR (500 MHz, acetone-*d*_6_) δ_H_ 7.04 (dq, *J* = 15.5, 6.9, Hz, H-3'), 6.98 (dq, *J* = 15.5, 6.9, Hz, H-3''), 5.97 (m, H-9), 5.85 (dq, *J* = 15.5, 1.7 Hz, H-2', H-2''), 5.74 (ddq, *J* = 15.4, 7.9, 1.6 Hz, H-8), 5.65 (dd, *J* = 4.4, 1.0 Hz, H-4), 4.96 (ddq, *J* = 7.9, 5.1, 0.9 Hz, H-2), 3.98 (dd, *J* = 5.1, 4.4 Hz, H-3), 1.90 (dd, *J* = 6.9, 1.7 Hz, 3H-4'), 1.88 (dd, *J* = 6.9, 1.7 Hz, 3H-4''), 1.79 (s, 3H-11), 1.74 (ddd, *J* = 6.6, 1.7, 0.8 Hz, 3H-10), 1.79 (s, 3H-11); ^13^C NMR (125 MHz, acetone-*d*_6_) δ_C_ 175.4 (C-7a), 167.1 (C-5), 165.7 (C-1''), 163.9 (C-1'), 149.1(C-3'), 146.7 (C-3''), 133.2 (C-9), 125.8 (C-8), 122.9 (C-2''), 122.3 (C-2'), 100.5 (C-7), 99.4 (C-4a), 86.0 (C-2), 69.8 (C-3), 65.1 (C-4), 23.2 (C-11), 18.2 (C-10), 18.1 (C-4', C-4''); HRESIMS (*m*/*z*): [M + H]^+^ calcd for C_19_H_23_O_8_^+^, 379.1387; found, 379.1383.

### Cytotoxic activity

The cytotoxicity against the murine fibroblasts L929, human cervix carcinoma KB-3-1, human lung carcinoma A549, human prostate cancer PC-3, epidermoid carcinoma A431, ovarian carcinoma SKOV-3, and breast cancer MCF-7 cell lines was determined by using the MTT (2-(4,5-dimethylthiazol-2-yl)-2,5-diphenyltetrazolium bromide) method in 96-well microplates as previously described [27–29]. Briefly, the cell lines were cultured in DMEM (Gibco), 60 µL aliquots of serial dilutions from an initial stock of 1 mg/mL in MeOH of the test compounds were added to 120 µL aliquots of a cell suspension (5 × 10^4^ cells/mL) in 96-well microplates. After 5 days incubation, an MTT assay was performed, and the absorbance measured at 590 nm using an ELISA plate reader (Victor). The concentration at which the growth of cells was inhibited to 50% of the control (IC_50_) was obtained from the dose–response curves. Epothilone B was used as the positive control.

### Nematicidal activity

Nematicidal activity was performed using *Caenorhabditis elegans* in a microtiter plate assay as described by Rupcic et al. (2018) [30]. The assay was performed in four concentrations (100, 50, 25 and 12.5 μg/mL). Ivermectin was used as a positive control at the same concentration ranges as the tested compounds and MeOH was used as a negative control. Percentages of mortality were calculated, then the results were expressed as a LD_90_ and LD_50_.

## Supporting Information

File 1 Copies of NMR, mass, and UV spectra for compounds **2**–**8**.

## Data Availability

All data is available in the published article and/or the supporting information.
