# Supplementary material for: Semisynthetic derivatives of massarilactone D with cytotoxic and nematicidal activities
Source: Beilstein J Org Chem. 2025 Mar 17;21:607–15. doi: 10.3762/bjoc.21.48 (PMC11931645; doi:10.3762/bjoc.21.48)
Supplement: File 1 — Copies of NMR, mass, and UV spectra for compounds 2–8. [file Beilstein_J_Org_Chem-21-607-s001.pdf]

BEILSTEIN JOURNAL OF ORGANIC CHEMISTRY

# Supporting Information

for

# Semisynthetic derivatives of massarilactone D with cytotoxic and nematicidal activities

Rémy B. Teponno, Sara R. Noumeur and Marc Stadler

*Beilstein J. Org. Chem.* **2025,** *21,* 607–615. doi:10.3762/bjoc.21.48

## Copies of NMR, mass, and UV spectra for compounds 2–8

License and Terms: This is a supporting information file under the terms of the Creative Commons Attribution License (https://creativecommons.org/licenses/by/4.0). Please note that the reuse, redistribution and reproduction in particular requires that the author(s) and source are credited and that individual graphics may be subject to special legal provisions.
The license is subject to the *Beilstein Journal of Organic Chemistry* terms and conditions: (https://www.beilstein-journals.org/bjoc/terms)

| | Contents | Page |
|---|---|---|
| **Figure S1**: | LC-UV-ESIMS spectra of compound **2** | S3 |
| **Figure S2**: | UV-HRESIMS spectra of compound **2** | S3 |
| **Figure S3**: | $^{1}$H NMR spectrum of compound **2** in acetone-$d_6$ at 500 MHz. | S4 |
| **Figure S4**: | $^{13}$C NMR spectrum of compound **2** in acetone-$d_6$ at 125 MHz. | S4 |
| **Figure S5**: | $^{1}$H-$^{1}$H COSY spectrum of compound **2** in acetone-$d_6$ | S5 |
| **Figure S6**: | HSQC spectrum of compound **2** in acetone-$d_6$ | S5 |
| **Figure S7**: | HMBC spectrum of compound **2** in acetone-$d_6$ | S6 |
| **Figure S8**: | ROESY spectrum of compound **2** in acetone-$d_6$ | S6 |
| **Figure S9**: | UV spectrum of compound **2** | S7 |
| **Figure S10**: | LC-UV-ESIMS spectra of compound **3** | S7 |
| **Figure S11**: | $^{1}$H NMR spectrum of compound **3** in acetone-$d_6$ at 700 MHz | S8 |
| **Figure S12**: | $^{1}$H NMR spectrum of compound **3** in acetone-$d_6$ at 175 MHz | S8 |
| **Figure S13**: | $^{1}$H-$^{1}$H COSY spectrum of compound **3** in acetone-$d_6$ | S9 |
| **Figure S14**: | HSQC spectrum of compound **3** in acetone-$d_6$ | S9 |
| **Figure S15**: | HMBC spectrum of compound **3** in acetone-$d_6$ | S10 |
| **Figure S16**: | ROESY spectrum of compound **3** in acetone-$d_6$ | S10 |
| **Figure S17**: | UV spectrum of compound **3** | S11 |
| **Figure S18**: | LC-UV-ESIMS spectra of compound **4** | S11 |
| **Figure S19**: | UV-HRESIMS spectra of compound 4 | S12 |
| **Figure S20**: | $^{1}$H NMR spectrum of compound **4** in acetone-$d_6$ at 700 MHz | S13 |
| **Figure S21**: | $^{13}$C NMR spectrum of compound **4** in acetone-$d_6$ at 175 MHz | S13 |
| **Figure S22**: | $^{1}$H-$^{1}$H COSY spectrum of compound **4** in acetone-$d_6$ | S14 |
| **Figure S23**: | HSQC spectrum of compound **4** in acetone-$d_6$ | S14 |
| **Figure S24**: | HMBC spectrum of compound **4** in acetone-$d_6$ | S15 |
| **Figure S25**: | ROESY spectrum of compound **4** in acetone-$d_6$ | S15 |
| **Figure S26**: | UV spectrum of compound **4** | S16 |
| **Figure S27**: | LC-UV-ESIMS spectra of compound **5** | S16 |

| | | |
|---|---|---|
| **Figure S28**: | $^1$H NMR spectrum of compound **5** in $CDCl_3$ at 500 MHz | S17 |
| **Figure S29**: | $^{13}$C NMR spectrum of compound **5** in $CDCl_3$ at 125 MHz | S17 |
| **Figure S30**: | $^1$H-$^1$H COSY spectrum of compound **5** | S18 |
| **Figure S31**: | HSQC spectrum of compound **5** | S18 |
| **Figure S32**: | HMBC spectrum of compound **5** | S19 |
| **Figure S33**: | ROESY spectrum of compound **5** | S19 |
| **Figure S34**: | UV spectrum of compound **5** | S20 |
| **Figure S35**: | LC-UV-ESIMS spectra of compound **6** | S20 |
| **Figure S36**: | UV-HRESIMS spectra of compound **6** | S21 |
| **Figure S37**: | $^1$H NMR spectrum of compound **6** in acetone-$d_6$ at 500 MHz | S21 |
| **Figure S38**: | $^{13}$C NMR spectrum of compound **6** in acetone-$d_6$ at 125 MHz | S22 |
| **Figure S39**: | $^1$H-$^1$H spectrum of compound **6** in acetone-$d_6$ | S22 |
| **Figure S40**: | HSQC spectrum of compound **6** in acetone-$d_6$ | S23 |
| **Figure S41**: | HMBC spectrum of compound **6** in acetone-$d_6$ | S23 |
| **Figure S42**: | ROESY spectrum of compound **6** in acetone-$d_6$ | S24 |
| **Figure S43**: | UV spectrum of compound **6** | S24 |
| **Figure S44**: | LC-UV-ESIMS spectrum of compound **7** | S25 |
| **Figure S44**: | UV-HRESIMS spectrum of compound **7** | S25 |
| **Figure S46**: | $^1$H NMR spectrum of compound **7** in acetone-$d_6$ at 500 MHz | S26 |
| **Figure S47**: | $^{13}$C NMR spectrum of compound **7** in acetone-$d_6$ at 125 MHz | S26 |
| **Figure S48**: | $^1$H-$^1$H COSY spectrum of compound **7** in acetone-$d_6$ | S27 |
| **Figure S49**: | HSQC spectrum of compound **7** in acetone-$d_6$ | S27 |
| **Figure S50**: | HMBC spectrum of compound **7** in acetone-$d_6$ | S28 |
| **Figure S51**: | ROESY spectrum of compound **7** in acetone-$d_6$ | S28 |
| **Figure S52**: | UV spectrum of compound **7** | S29 |
| **Figure S53**: | LC-UV-HRESIMS spectrum of compound **8** | S29 |
| **Figure S54**: | UV-HRESIMS spectrum of compound **8** | S30 |
| **Figure S55**: | $^1$H NMR spectrum of compound **8** in acetone-$d_6$ at 500 MHz | S30 |

| | | |
|---|---|---|
| **Figure S56**: | $^{13}C$ NMR spectrum of compound **8** in acetone-$d_6$ at 125 MHz | S31 |
| **Figure S57**: | $^{1}H$-$^{1}H$ COSY spectrum of compound **8** in acetone-$d_6$ | S31 |
| **Figure S58**: | HSQC spectrum of compound **8** in acetone-$d_6$ | S32 |
| **Figure S59**: | HMBC spectrum of compound **8** in acetone-$d_6$ | S32 |
| **Figure S60**: | ROESY spectrum of compound **8** in acetone-$d_6$ | S33 |
| **Figure S61**: | UV spectrum of compound **8** | S33 |
| **Figure S62**: | Chemical structures of rosigenin, curvupallides A-C, and spirostaphylotrichin X | S34 |


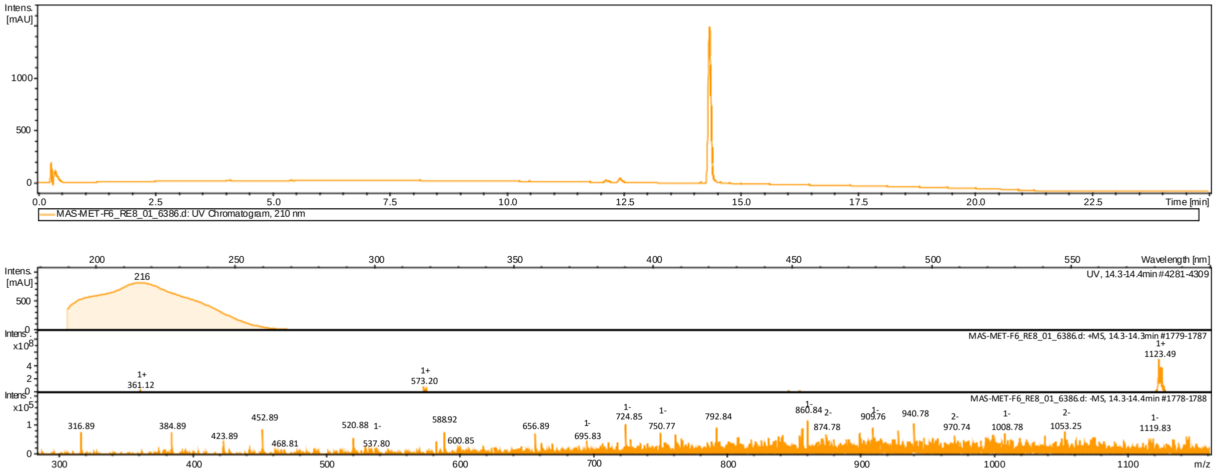


**Figure S1**: LC-UV-ESIMS spectra of compound **2**

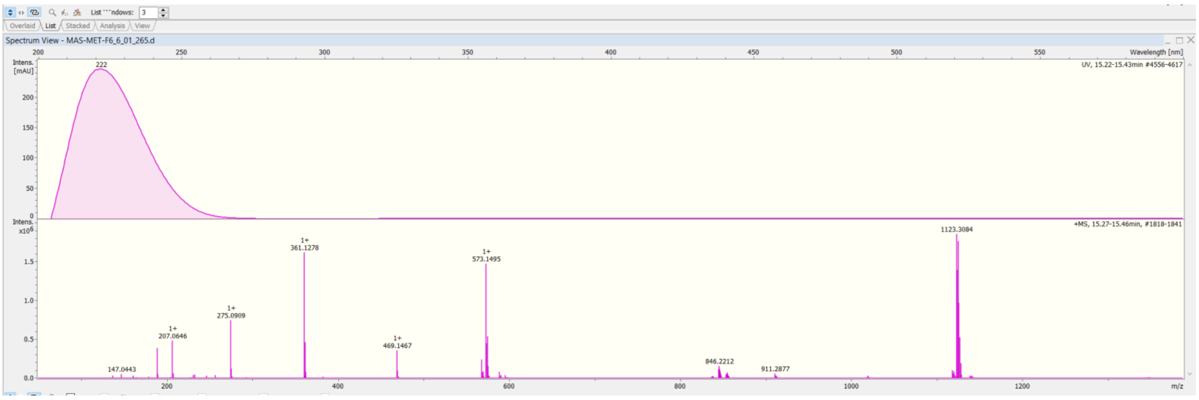


**Figure S2**: UV-HRESIMS spectra of compound **2**.

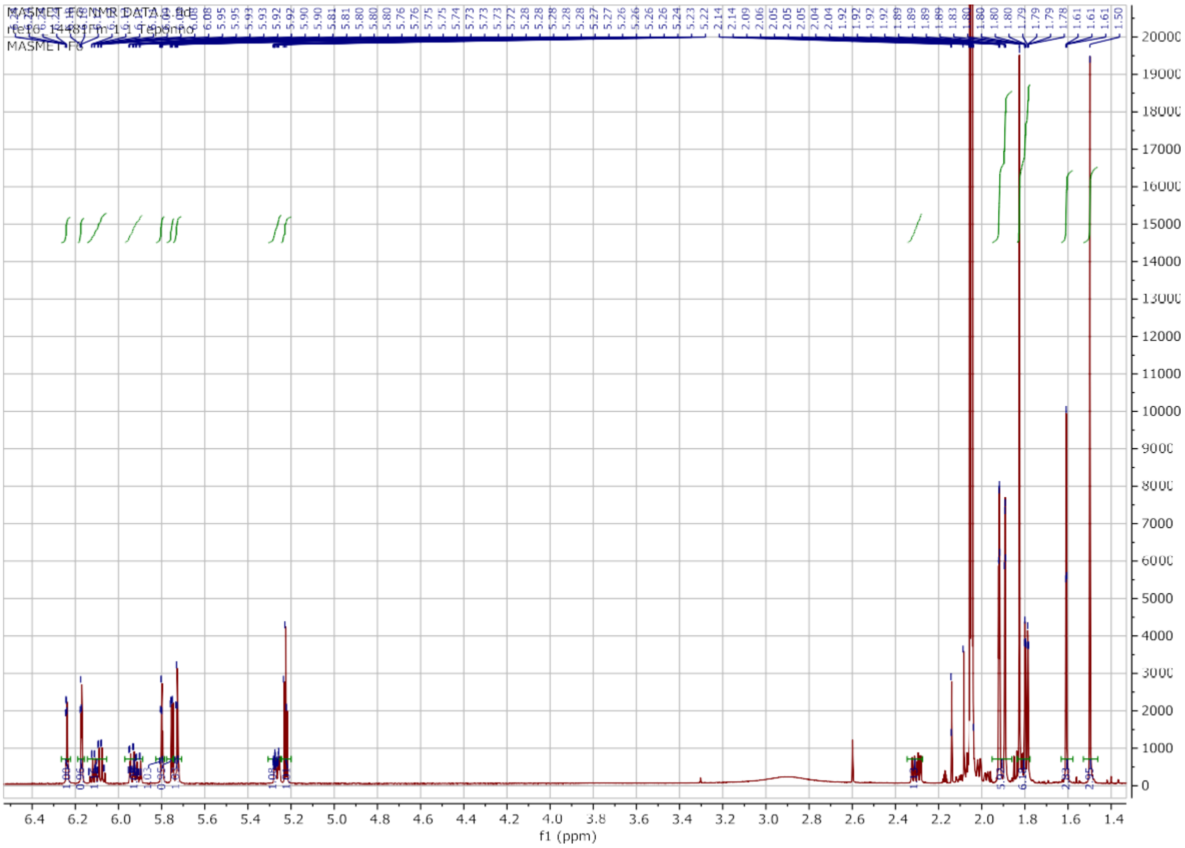

**Figure S3**: $^{1}H$ NMR spectrum of compound **2** in acetone-$d_6$ at 500 MHz.

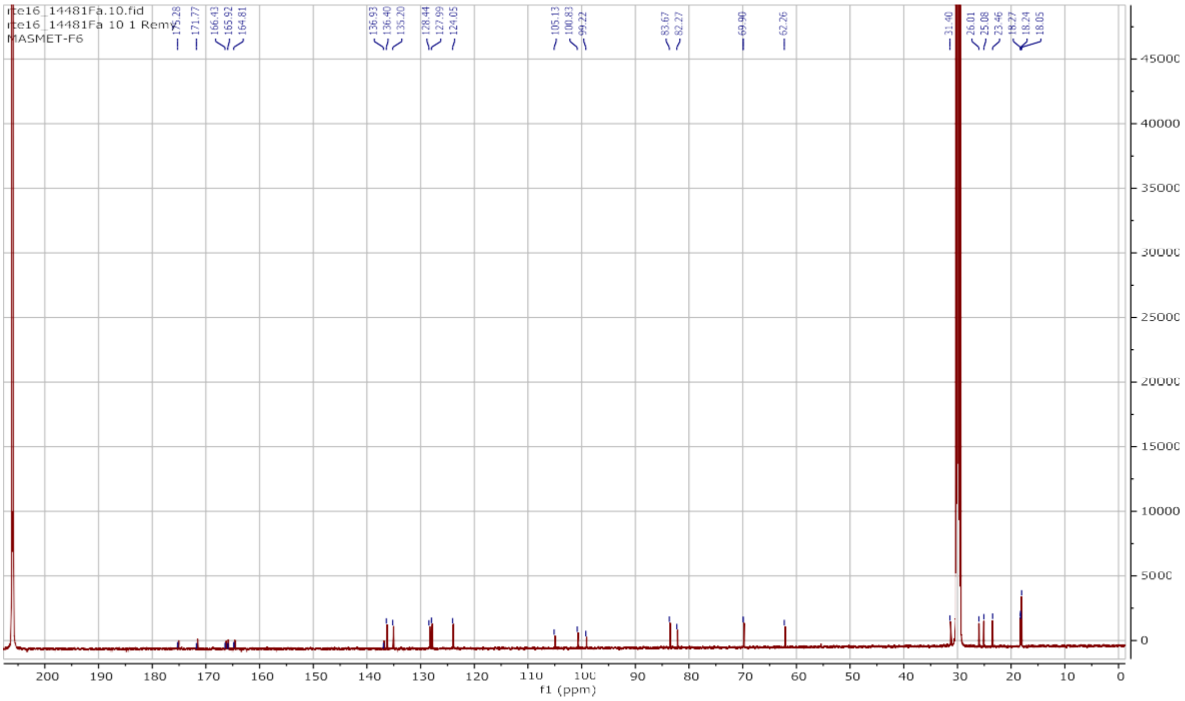

**Figure S4**: $^{13}C$ NMR spectrum of compound **2** in acetone-$d_6$ at 125 MHz.

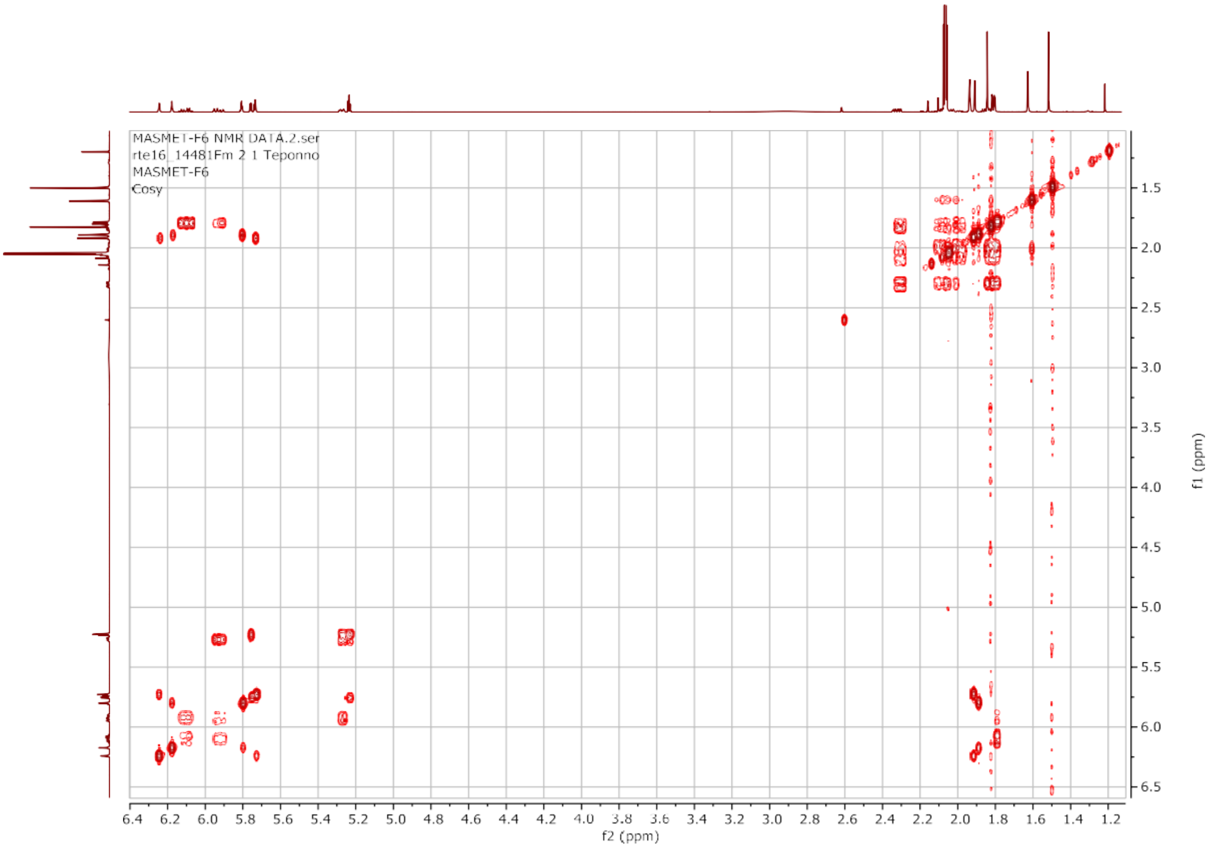


**Figure S5**: $^{1}H$-$^{1}H$ COSY spectrum of compound **2** in acetone-$d_6$

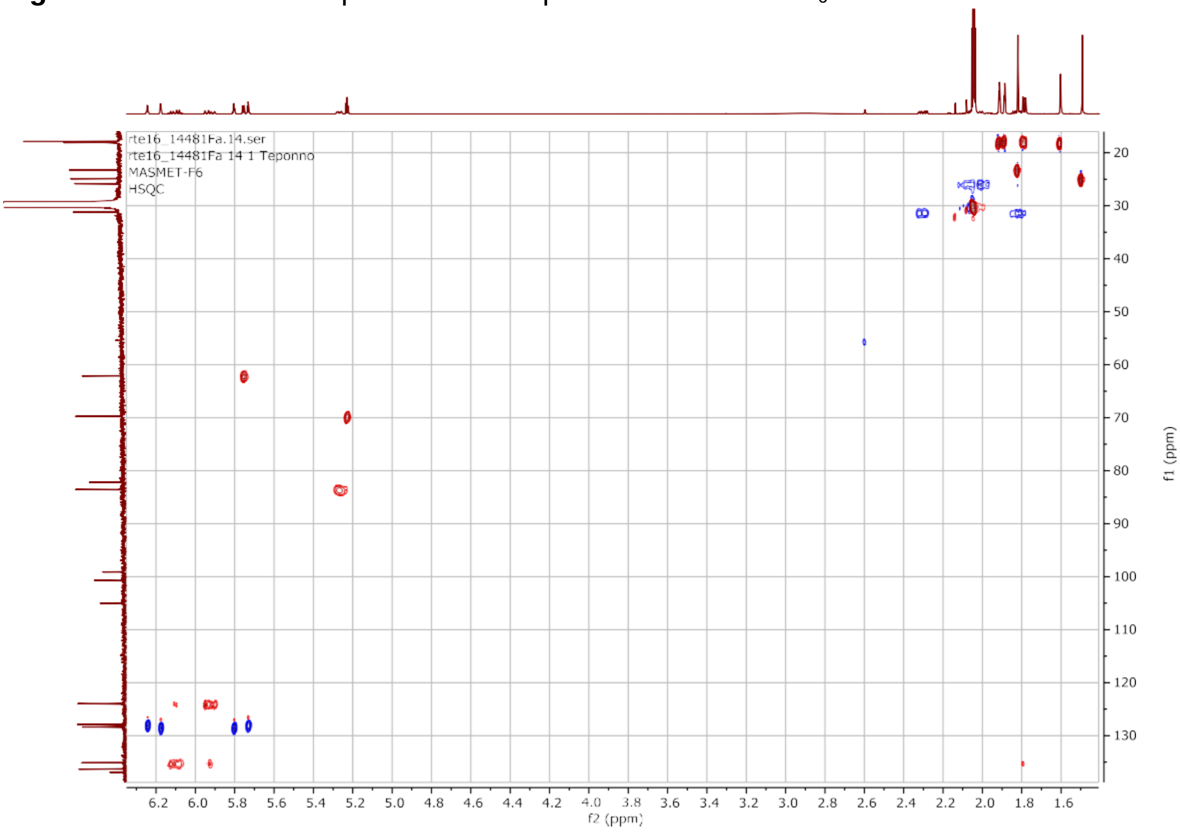


**Figure S6**: HSQC spectrum of compound **2** in acetone-$d_6$

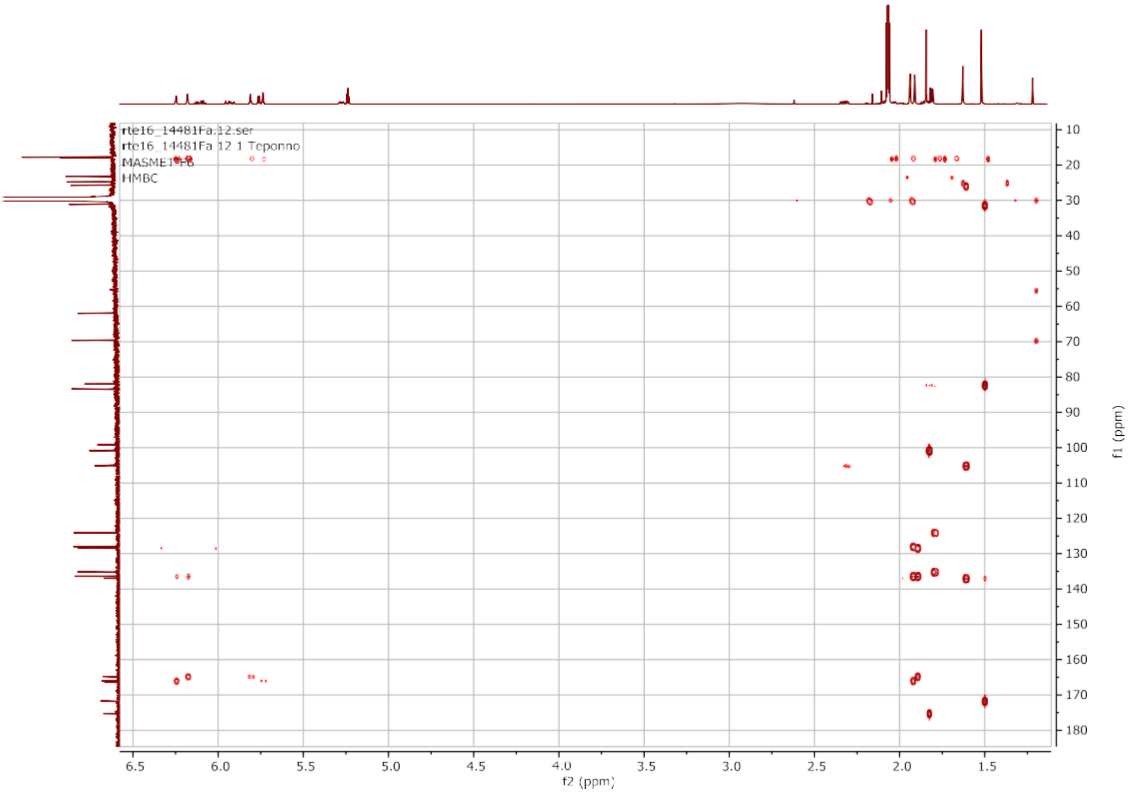


**Figure S7**: HMBC spectrum of compound **2** in acetone-$d_6$

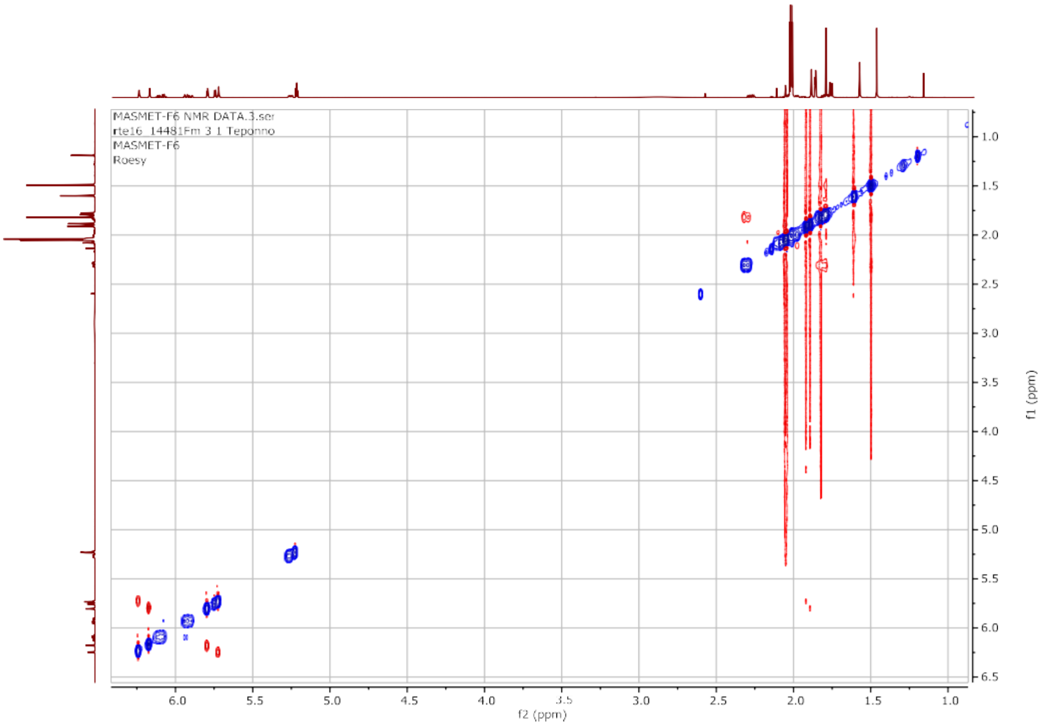


**Figure S8**: ROESY spectrum of compound **2** in acetone-$d_6$

## Spectrum Peak Pick Report

20.04.2018 13:13:50

Data Set: MAS-MET-F6-0.075 - RawData

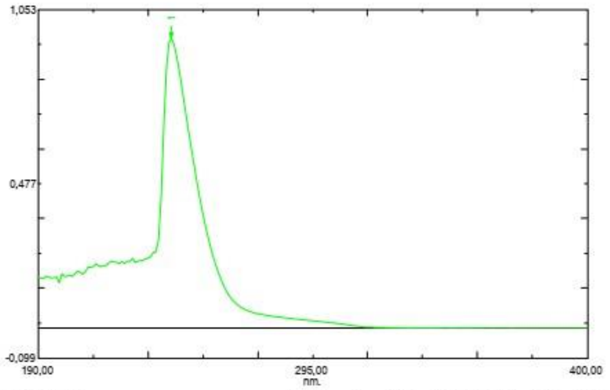


| No. | P/V | Wavelength | Abs. | Description |
|---|---|---|---|---|
| 1 | | 241,00 | 0,957 | |
| 2 | | 361,00 | -0,003 | |

Measurement Properties
Wavelength Range (nm.): 190,00 to 400,00
Scan Speed: Medium
Sampling Interval: 1,0
Auto Sampling Interval: Disabled
Scan Mode: Single

Instrument Properties
Instrument Type: UV-2400PC Series
Measuring Mode: Absorbance
Slit Width: 2,0 nm
Light Source Change Wavelength: 360,0 nm
S/R Exchange: Normal

Attachment Properties
Attachment: None

Sample Preparation Properties
Weight: 0.075
Volume: 1mL
Dilution:
Path Length: 1 cm
Additional Information: MAS-MET-F6-0.075

**Figure S9**: UV spectrum of compound **2**

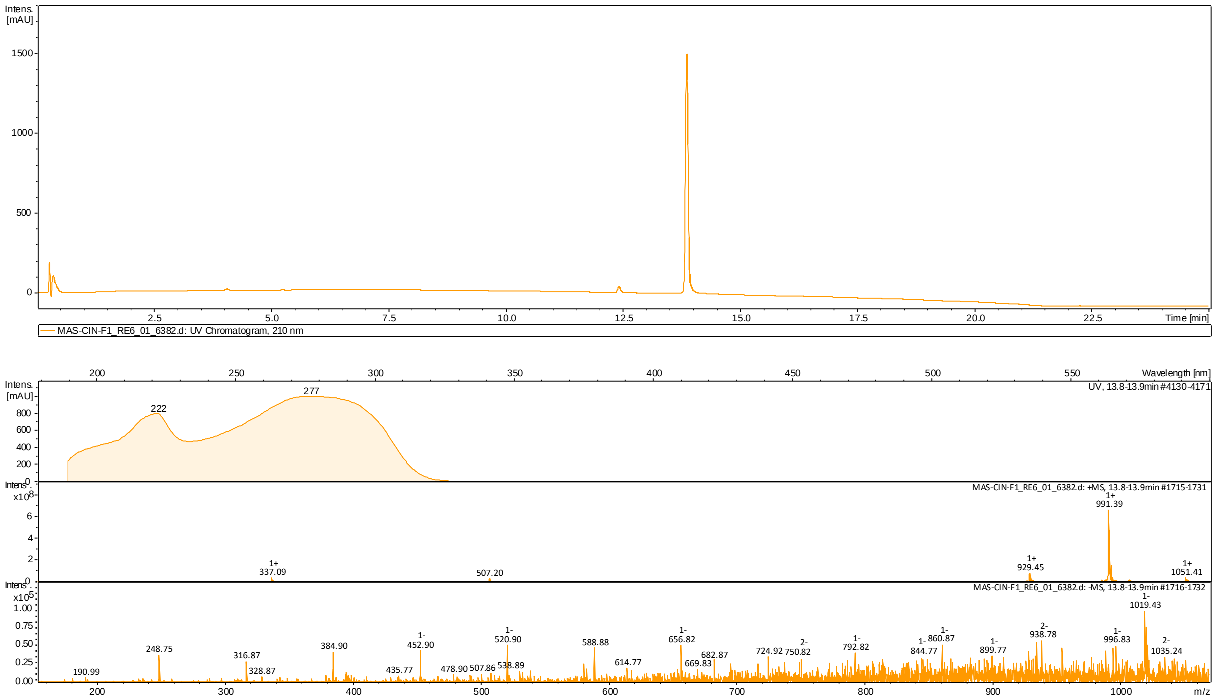


**Figure S10**: LC-UV-ESIMS spectra of compound **3**.

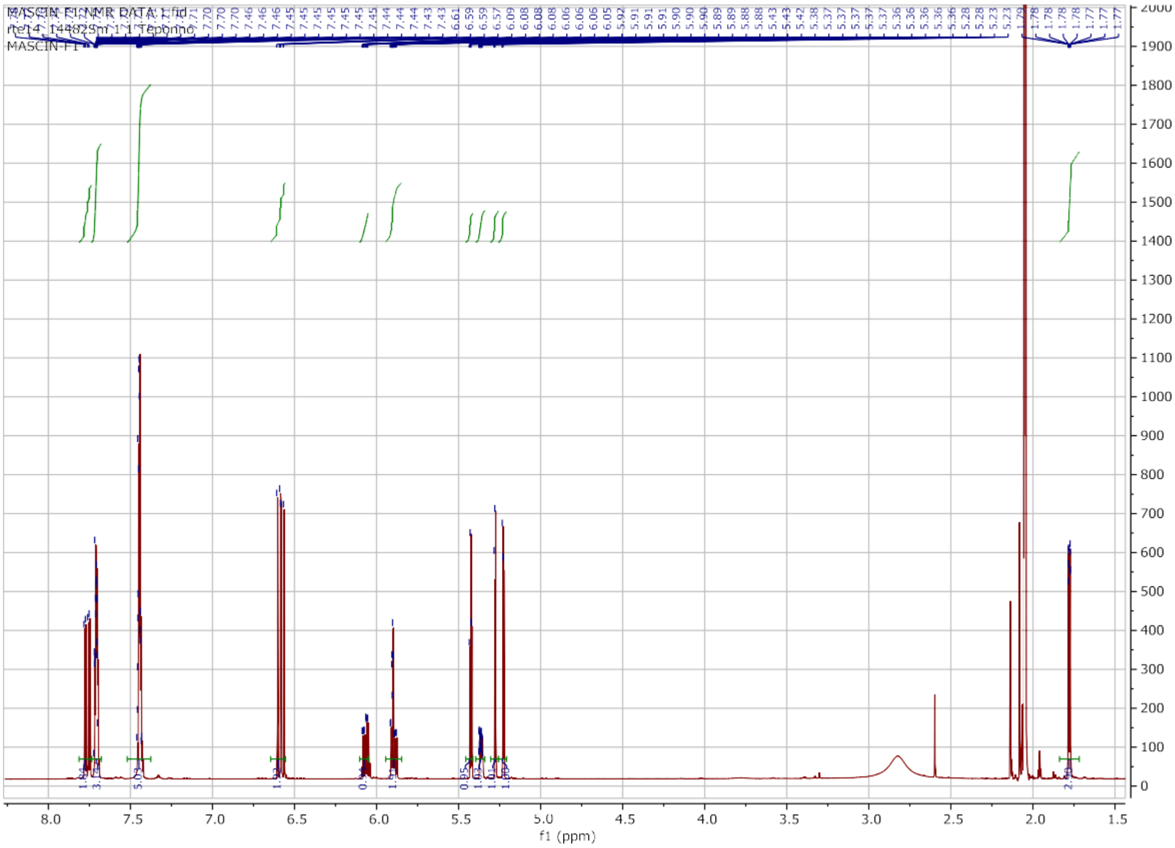

**Figure S11**: $^{1}$H NMR spectrum of compound **3** in acetone-$d_6$ at 700 MHz.

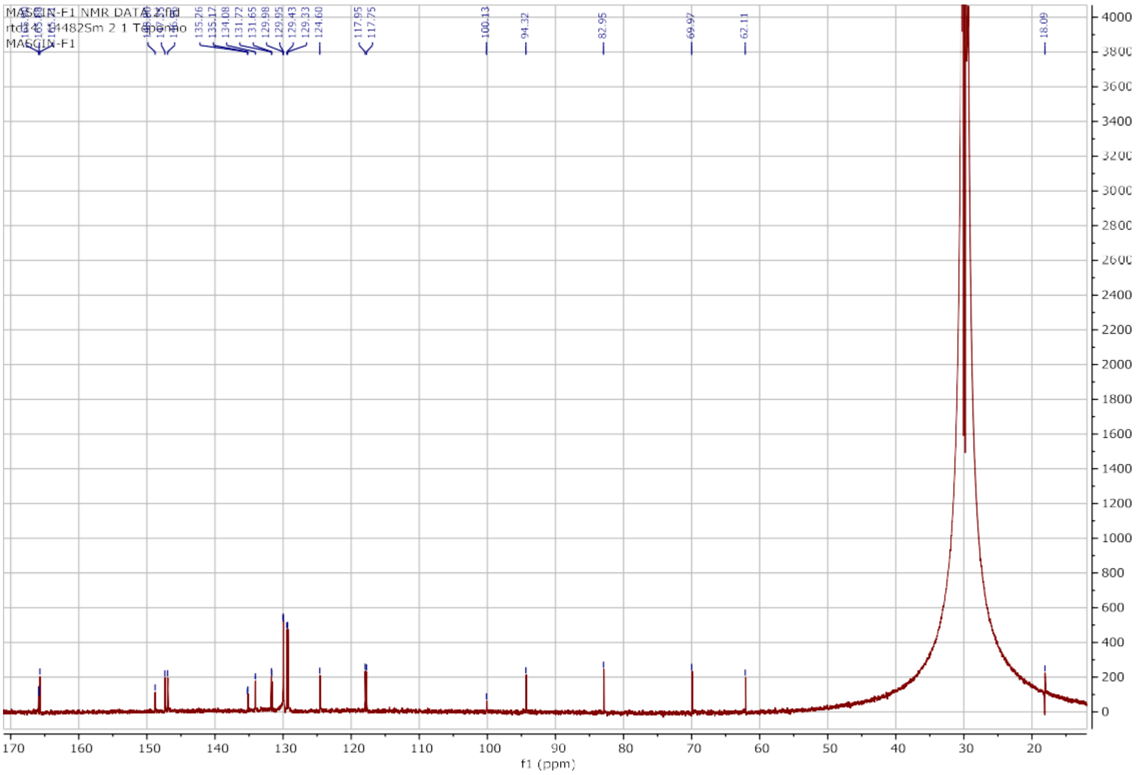

**Figure S12**: $^{1}$H NMR spectrum of compound **3** in acetone-$d_6$ at 175 MHz.

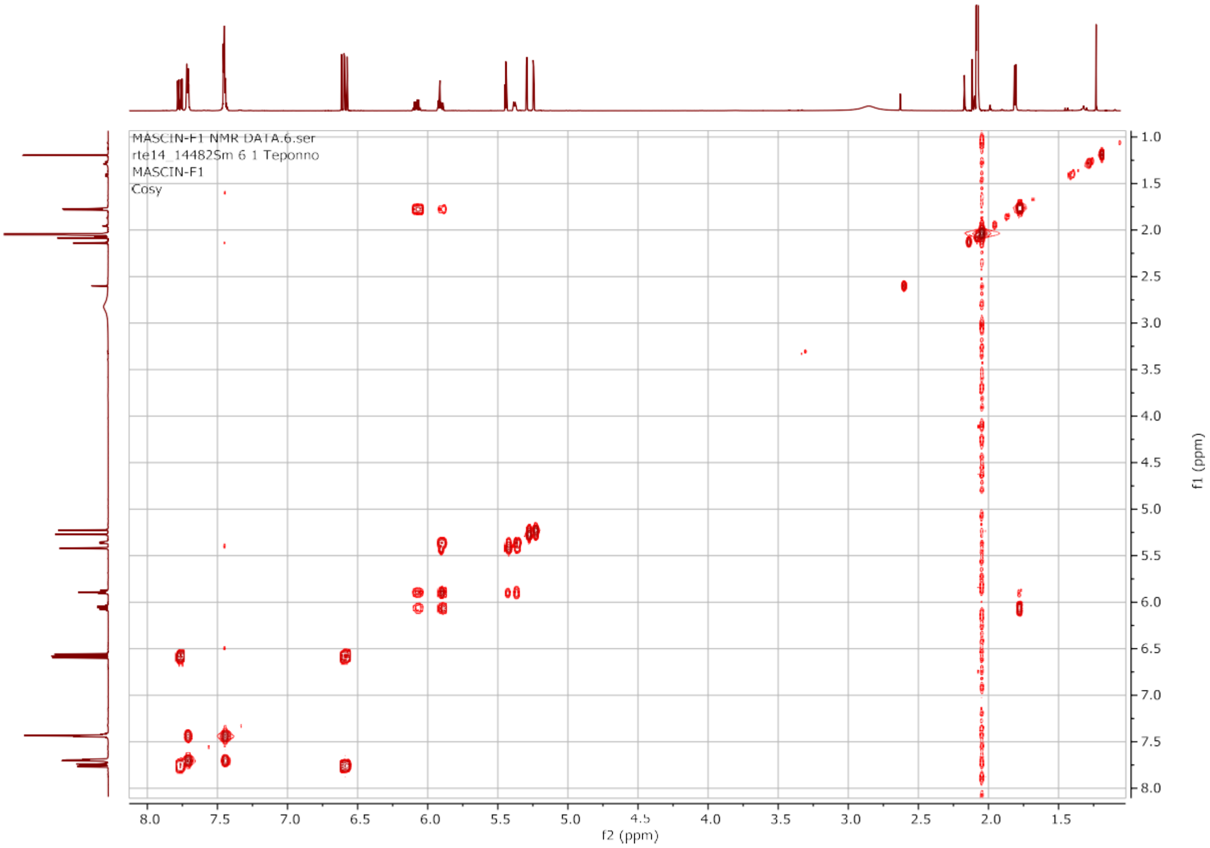


**Figure S13**: $^{1}H$-$^{1}H$ COSY spectrum of compound **3** in acetone-$d_6$

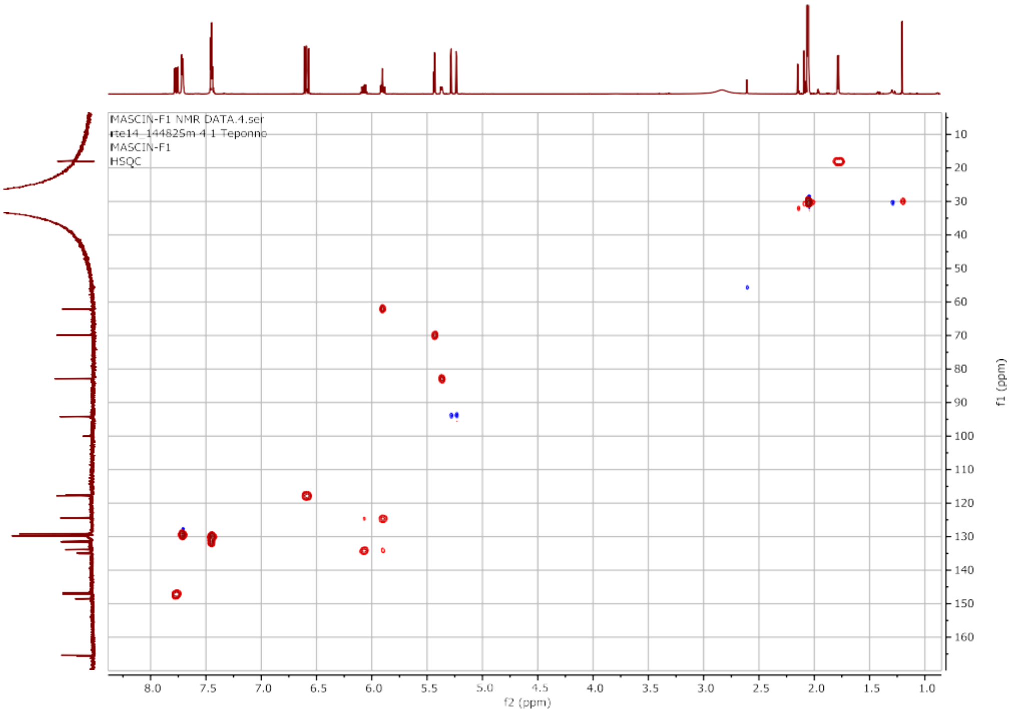


**Figure S14**: HSQC spectrum of compound **3** in acetone-$d_6$

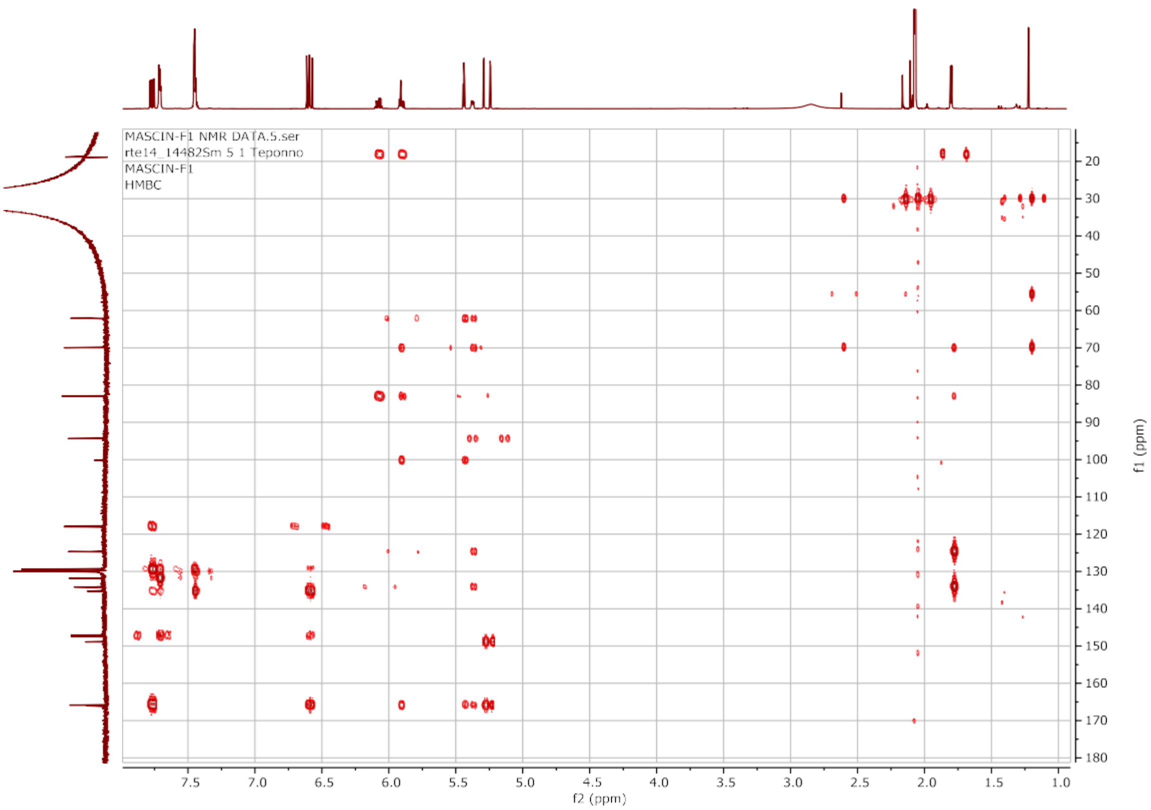


**Figure S15**: HMBC spectrum of compound **3** in acetone-$d_6$

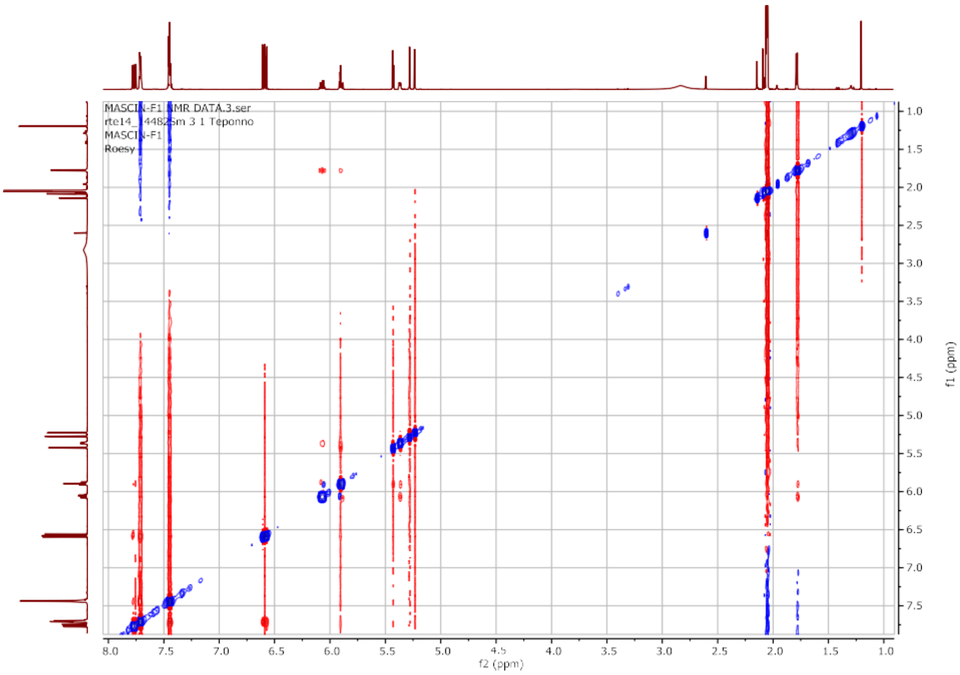


**Figure S16**: ROESY spectrum of compound **3** in acetone-$d_6$

**Spectrum Peak Pick Report** 20.04.2018 13:36:06

Data Set: MAS-CIN-F1-0.01875 - RawData

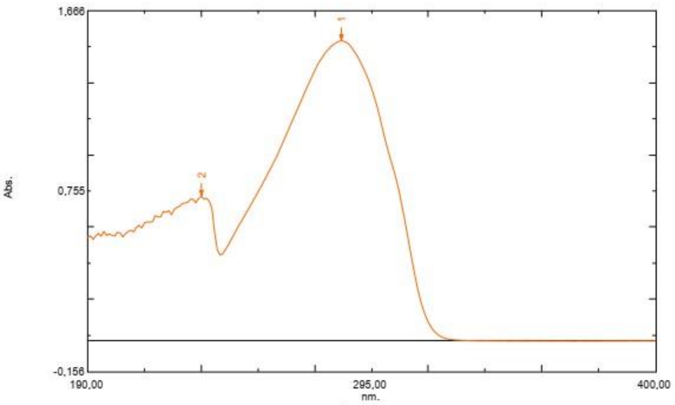


| No. | P/V | Wavelength | Abs. | Description |
|---|---|---|---|---|
| 1 | ↑ | 284,00 | 1,514 | |
| 2 | ↑ | 232,00 | 0,728 | |
| 3 | ↓ | 239,00 | 0,431 | |

| | |
|---|---|
| Measurement Properties | |
| Wavelength Range (nm.): | 190,00 to 400,00 |
| Scan Speed: | Medium |
| Sampling Interval: | 1,0 |
| Auto Sampling Interval: | Disabled |
| Scan Mode: | Single |
| Instrument Properties | |
| Instrument Type: | UV-2400PC Series |
| Measuring Mode: | Absorbance |
| Slit Width: | 2,0 nm |
| Light Source Change Wavelength: | 360,0 nm |
| S/R Exchange: | Normal |
| Attachment Properties | |
| Attachment: | None |
| Sample Preparation Properties | |
| Weight: | 0.01875 |
| Volume: | 1mL |
| Dilution: | |
| Path Length: | 1 cm |
| Additional Information: | MAS-CIN-F1-0.01875 |

**Figure S17**: UV spectrum of compound **3**

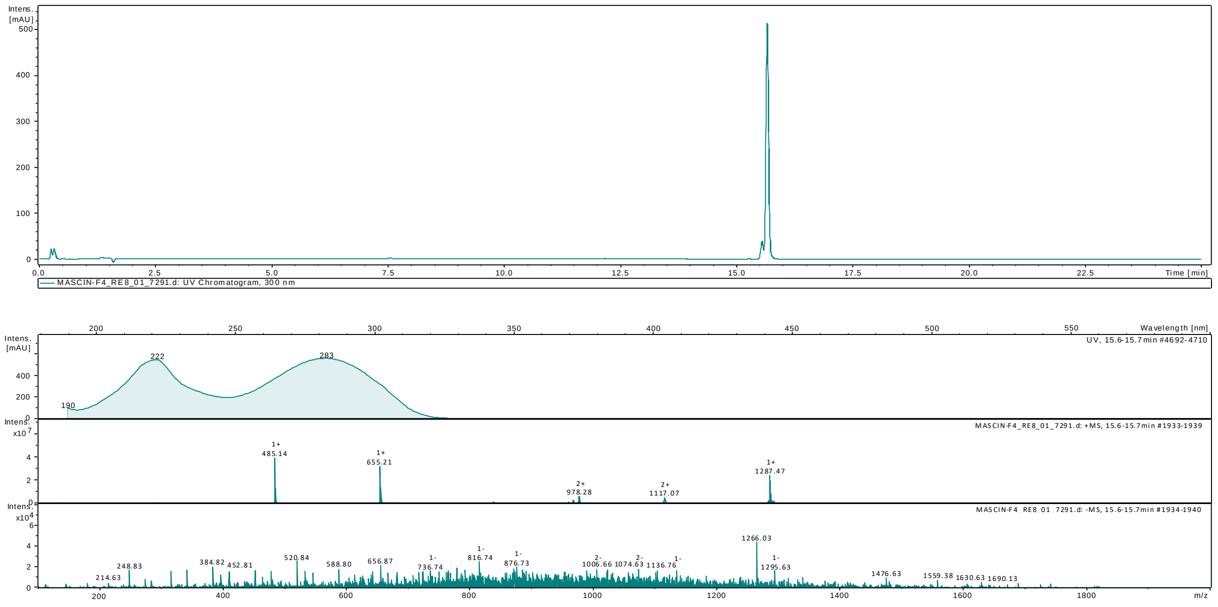


**Figure S18**: LC-UV-ESIMS spectra of compound **4**

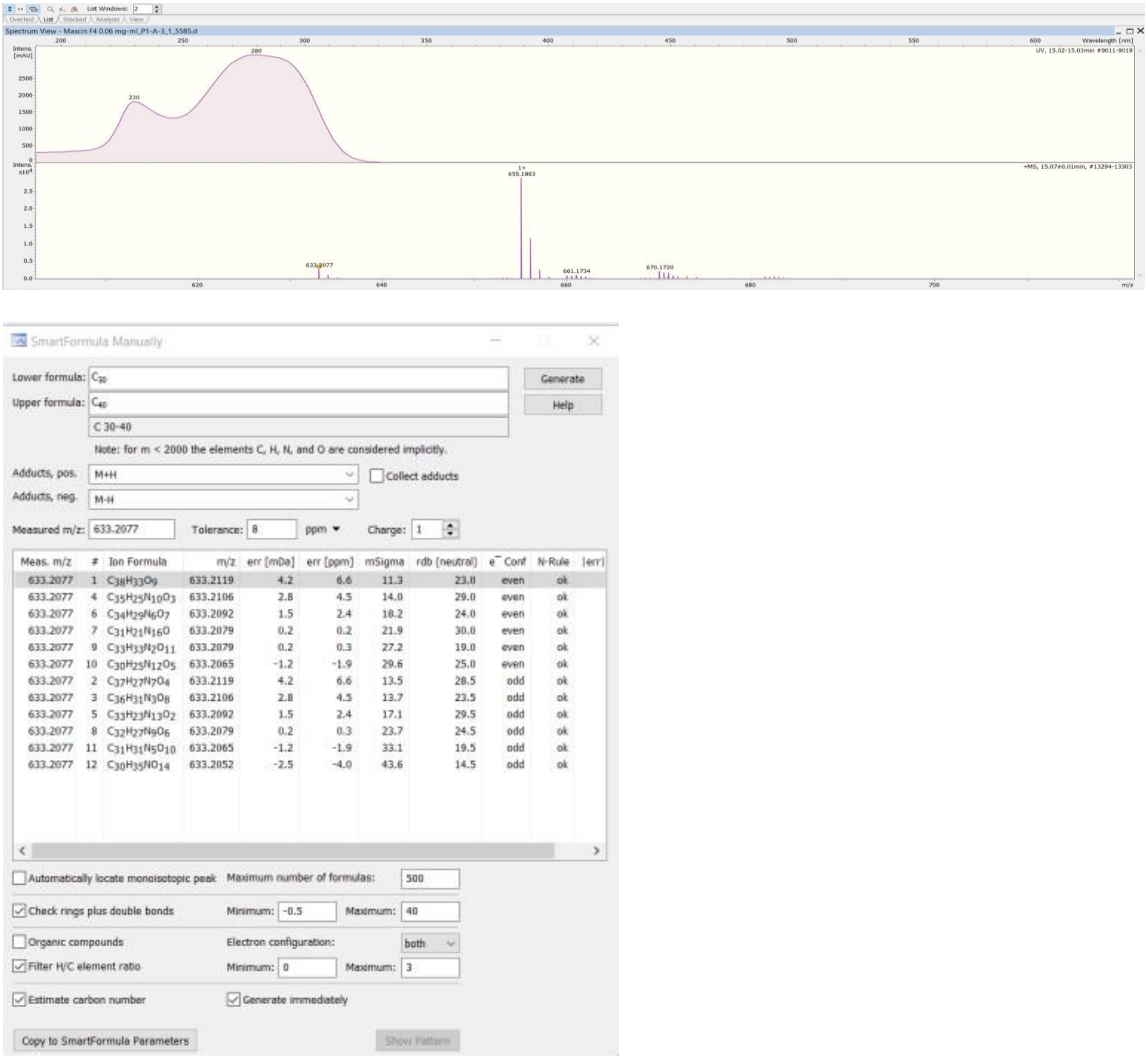


**Figure S19**: UV-HRESIMS spectra of compound **4**.

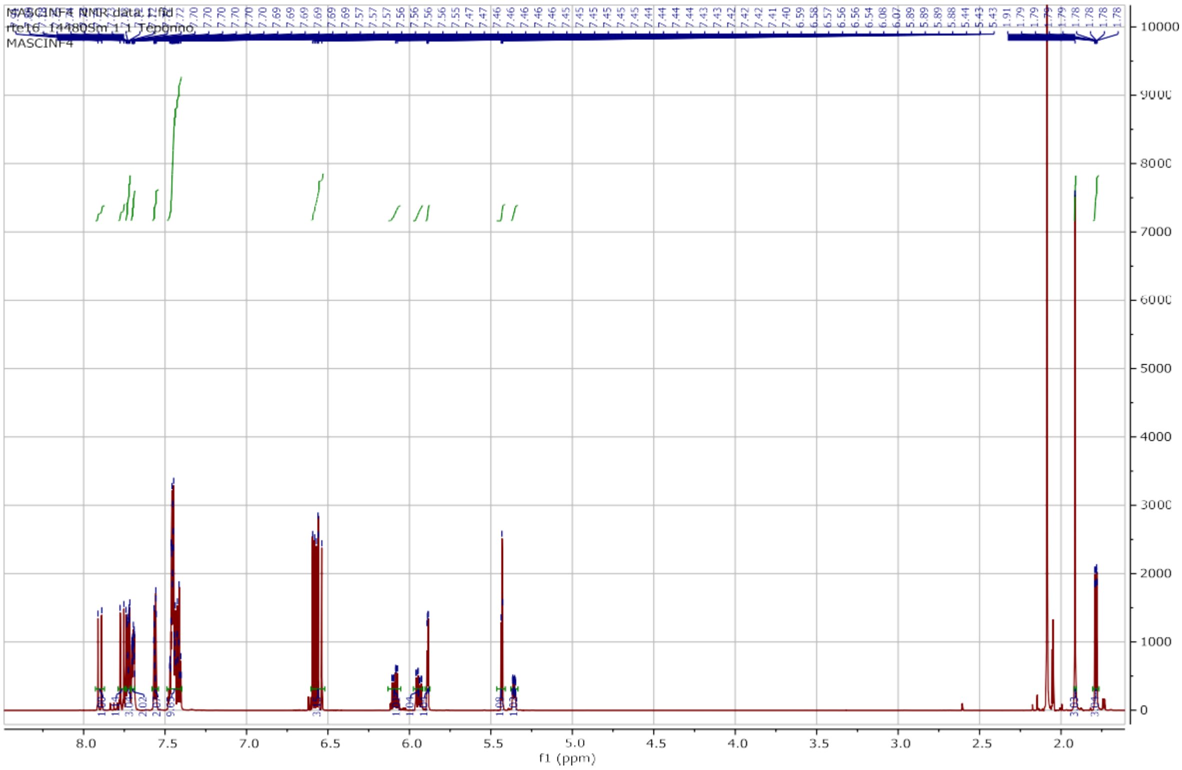

**Figure S20**: $^{1}H$ NMR spectrum of compound **4** in acetone-$d_6$ at 700 MHz.

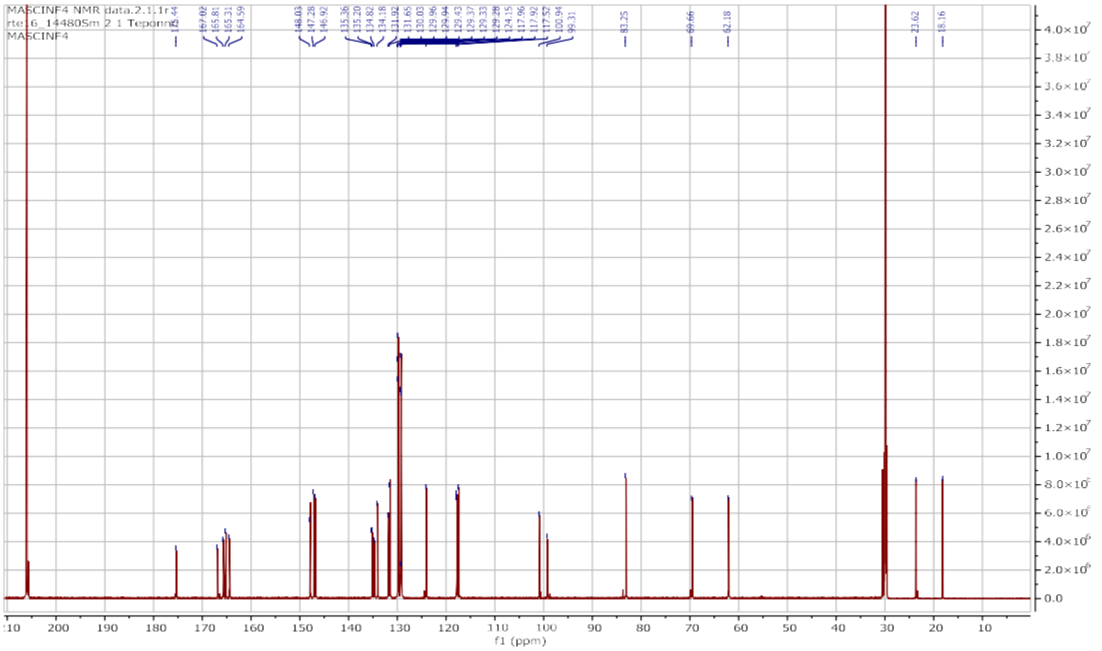

**Figure S21**: $^{13}C$ NMR spectrum of compound **4** in acetone-$d_6$ at 175 MHz.

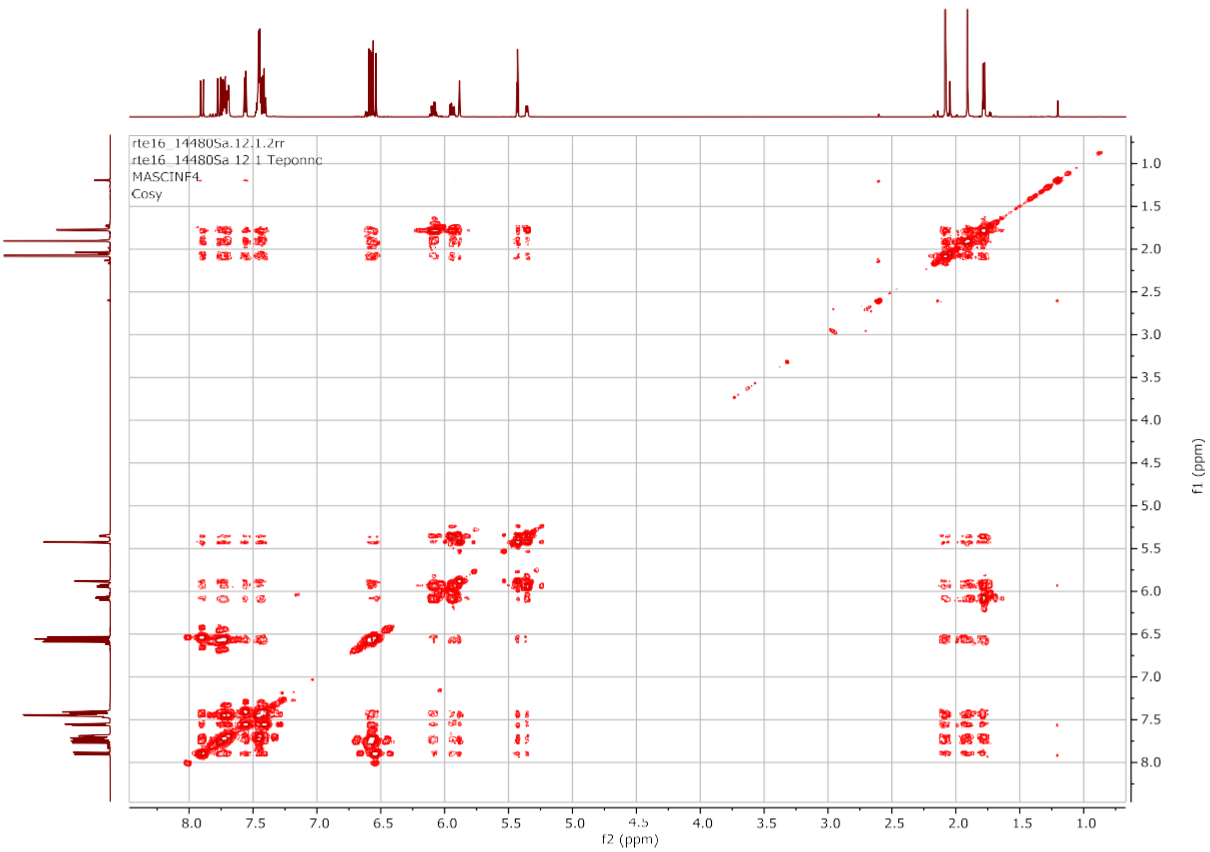


**Figure S22**: $^1H$-$^1H$ COSY spectrum of compound **4** in acetone-$d_6$.

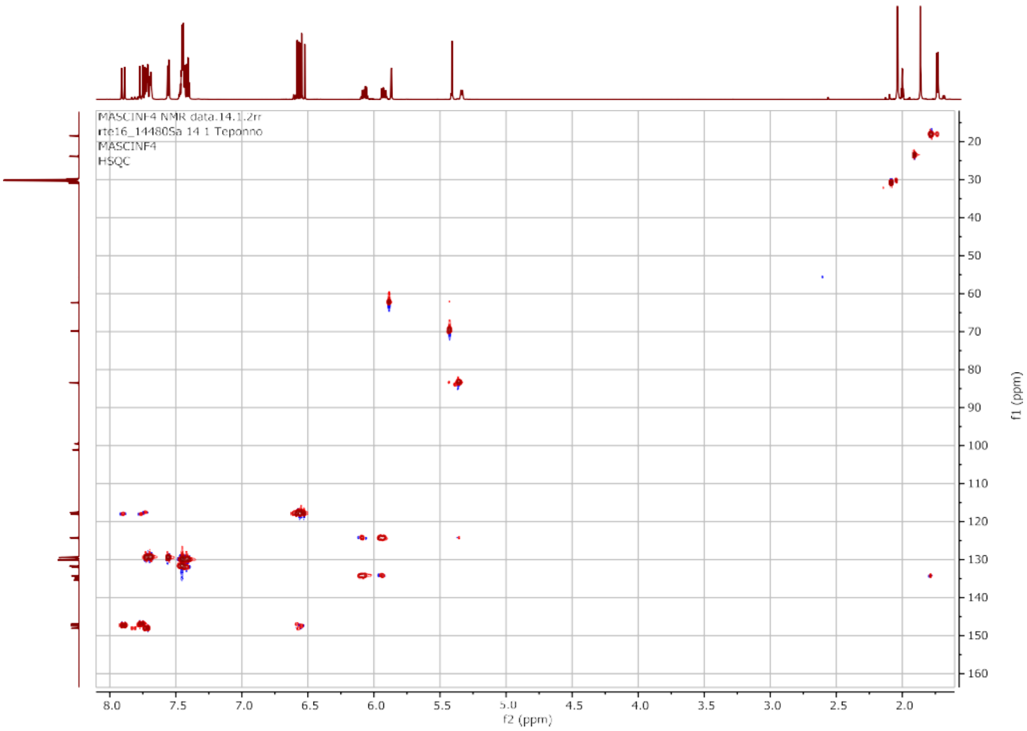


**Figure S23**: HSQC spectrum of compound **4** in acetone-$d_6$.

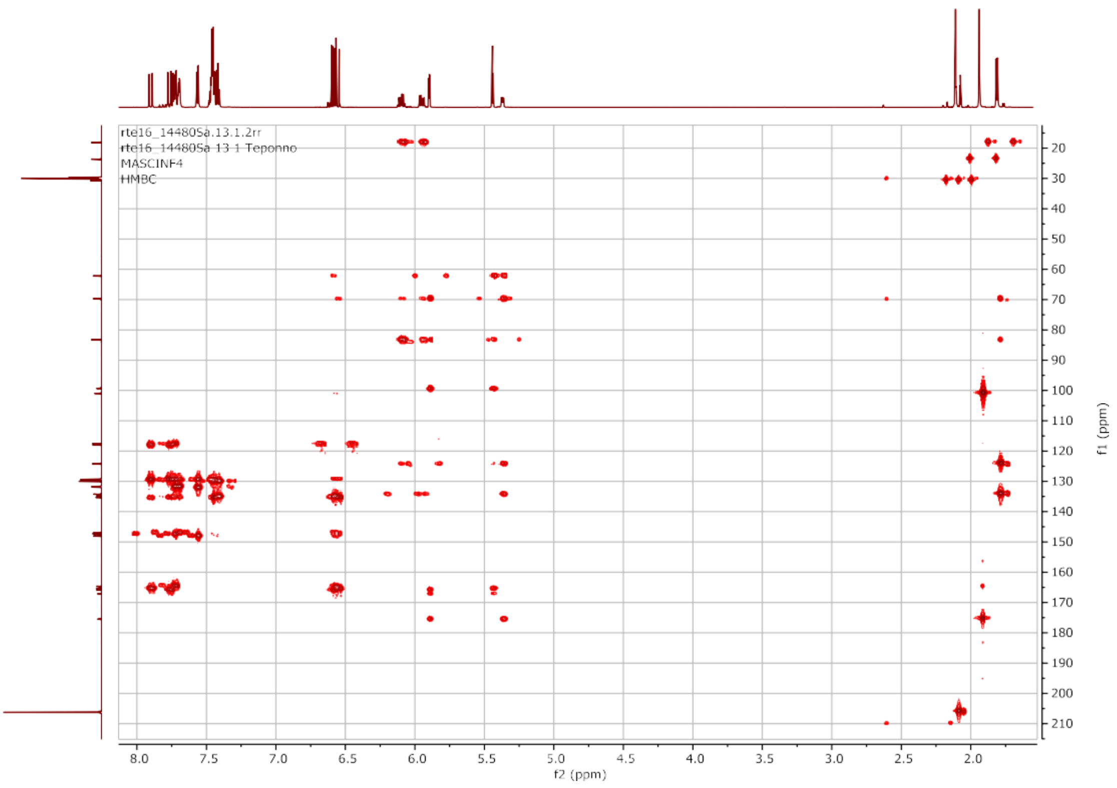


**Figure S24**: HMBC spectrum of compound **4** in acetone-$d_6$.

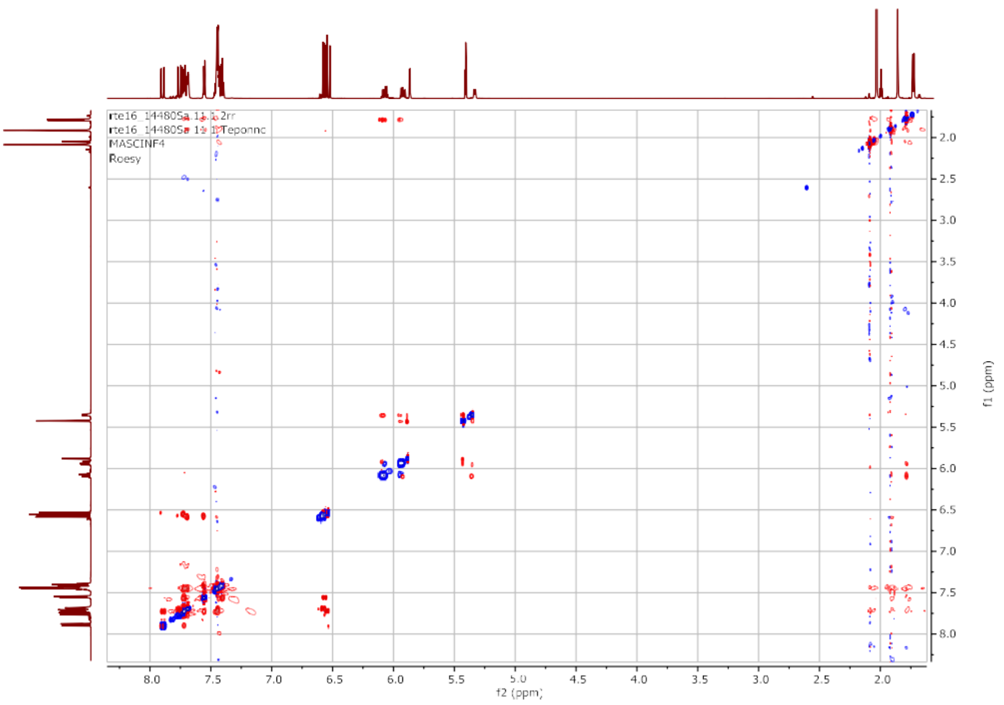


**Figure S25**: ROESY spectrum of compound **4** in acetone-$d_6$.

## Spectrum Peak Pick Report

20.04.2018 13:28:09

Data Set: MAS-CIN-F2-0.009375 - RawData

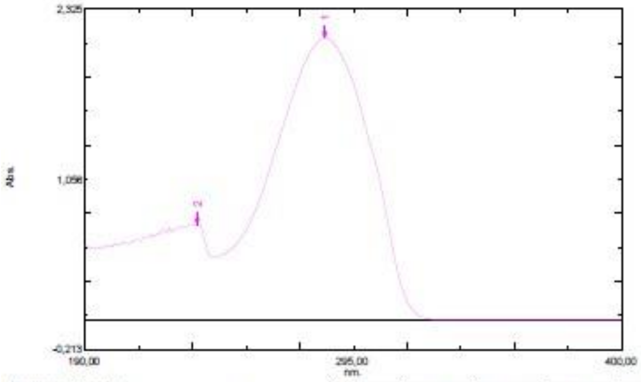


| No. | P/V | Wavelength | Abs. | Description |
|---|---|---|---|---|
| 1 | P | 284,00 | 2,113 | |
| 2 | P | 234,00 | 0,724 | |
| 3 | V | 241,00 | 0,472 | |

| | |
|---|---|
| Measurement Properties | |
| Wavelength Range (nm.): | 190,00 to 400,00 |
| Scan Speed: | Medium |
| Sampling Interval: | 1,0 |
| Auto Sampling Interval: | Disabled |
| Scan Mode: | Single |
| Instrument Properties | |
| Instrument Type: | UV-2400PC Series |
| Measuring Mode: | Absorbance |
| Slit Width: | 2,0 nm |
| Light Source Change Wavelength: | 360,0 nm |
| S/R Exchange: | Normal |
| Attachment Properties | |
| Attachment: | None |
| Sample Preparation Properties | |
| Weight: | 0.009375 |
| Volume: | 1mL |
| Dilution: | |
| Path Length: | 1 cm |
| Additional Information: | MAS-CIN-F2-0.009375 |

**Figure S26**: UV spectrum of compound **4** in acetone-$d_6$.

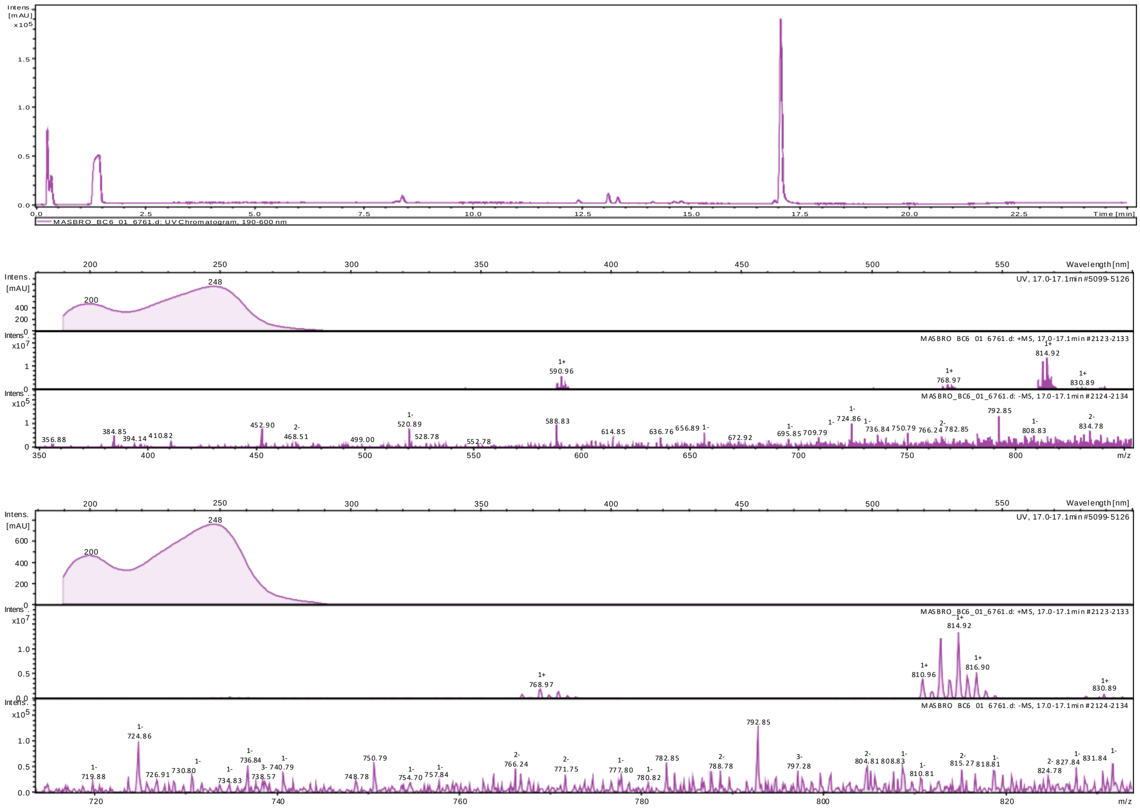


**Figure S27**: LC-UV-ESIMS spectra of compound **5**.

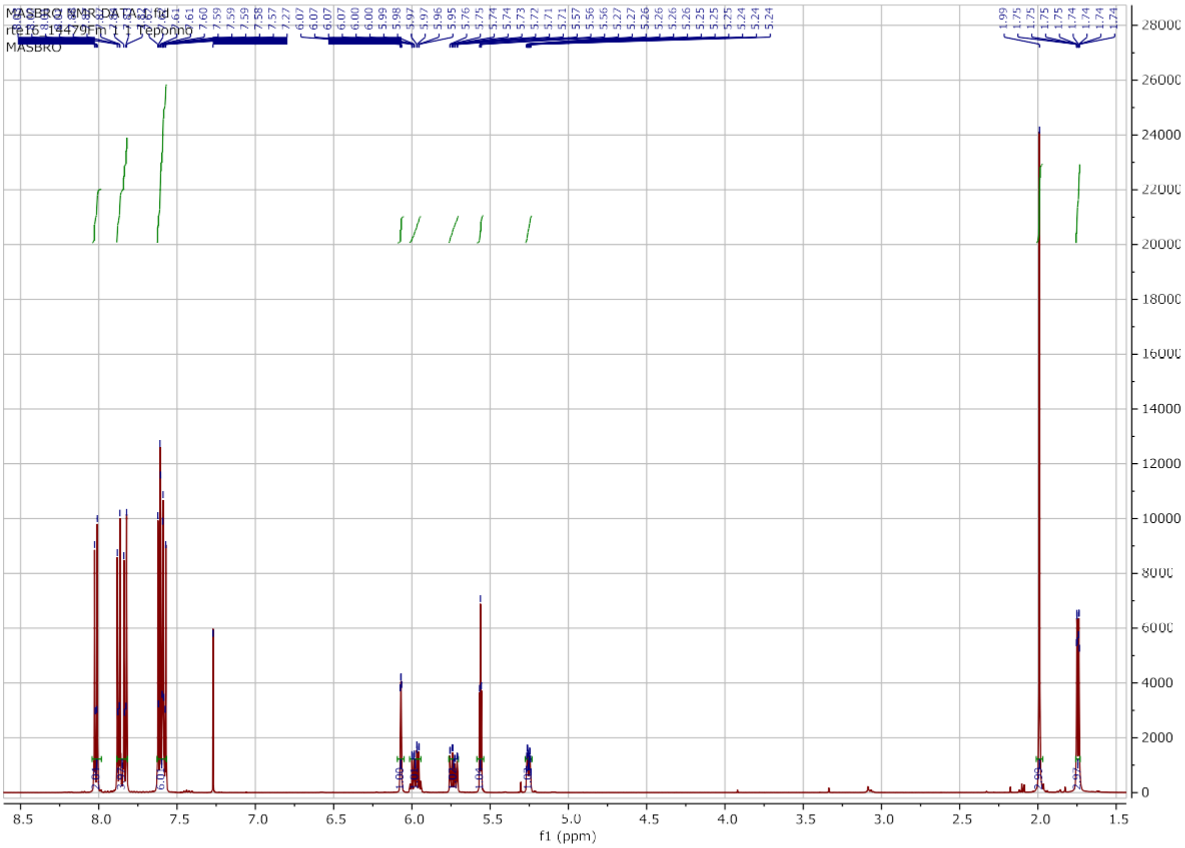


**Figure S28**: $^{1}$H NMR spectrum of compound **5** in $CDCl_3$ at 500 MHz.

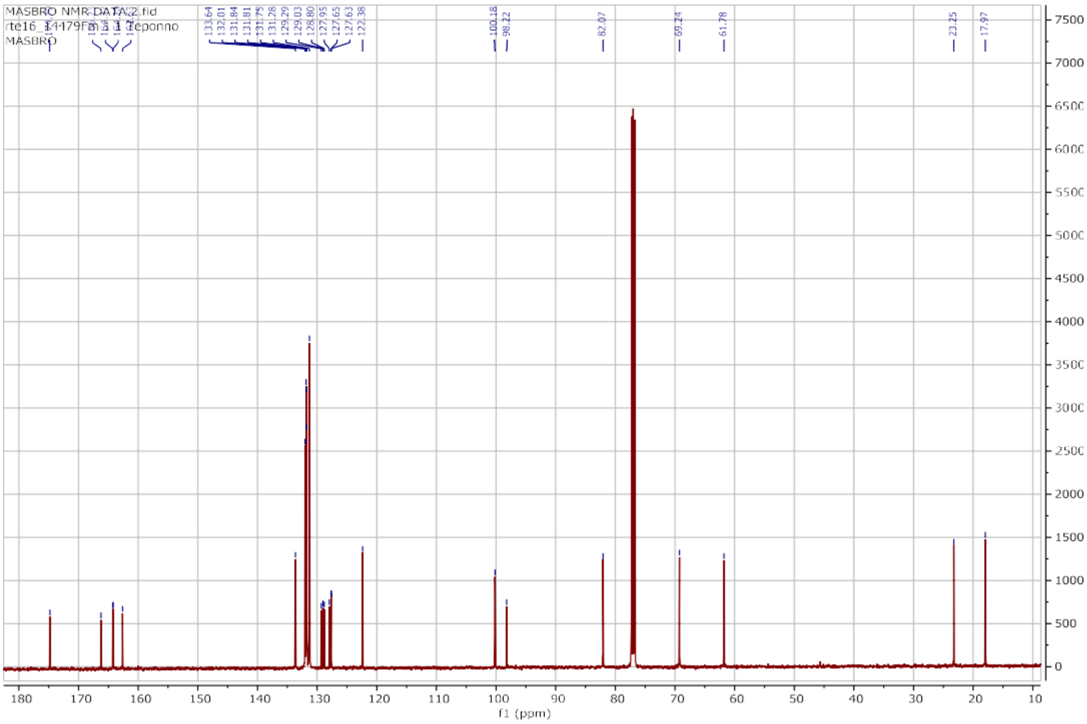


**Figure S29**: $^{13}$C NMR spectrum of compound **5** in $CDCl_3$ at 125 MHz.

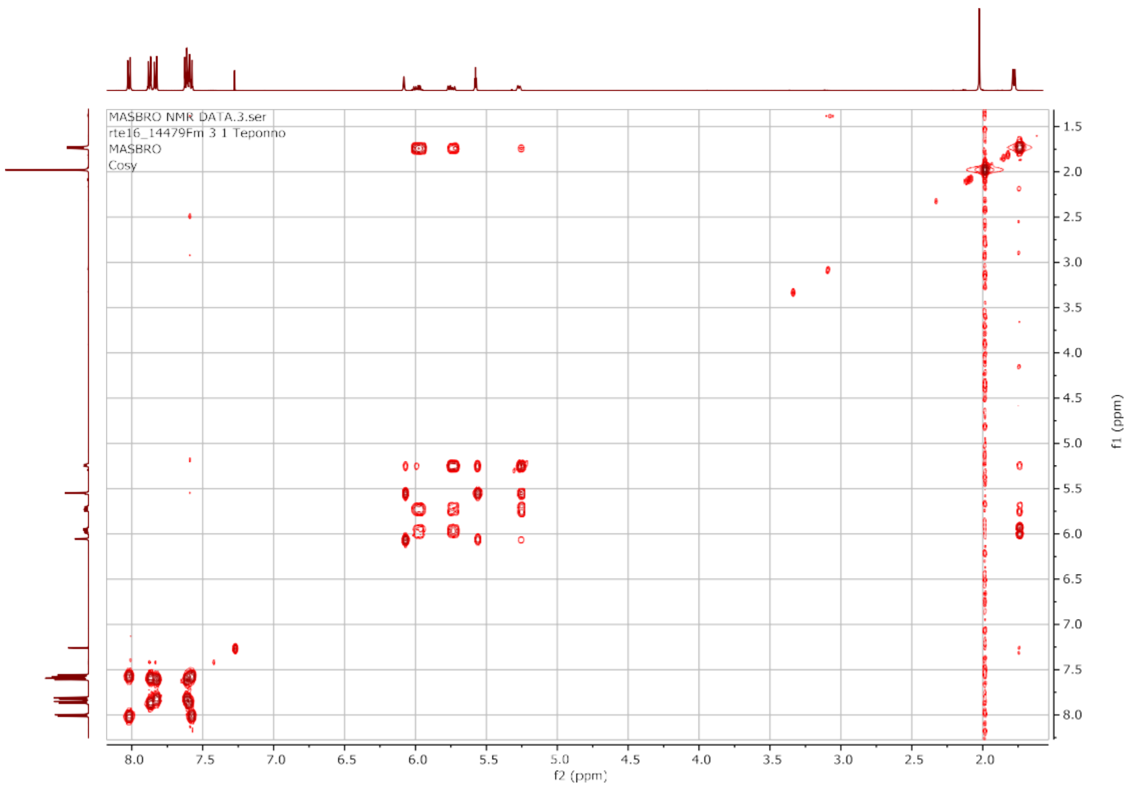


**Figure S30**: $^{1}H$-$^{1}H$ COSY spectrum of compound **5**.

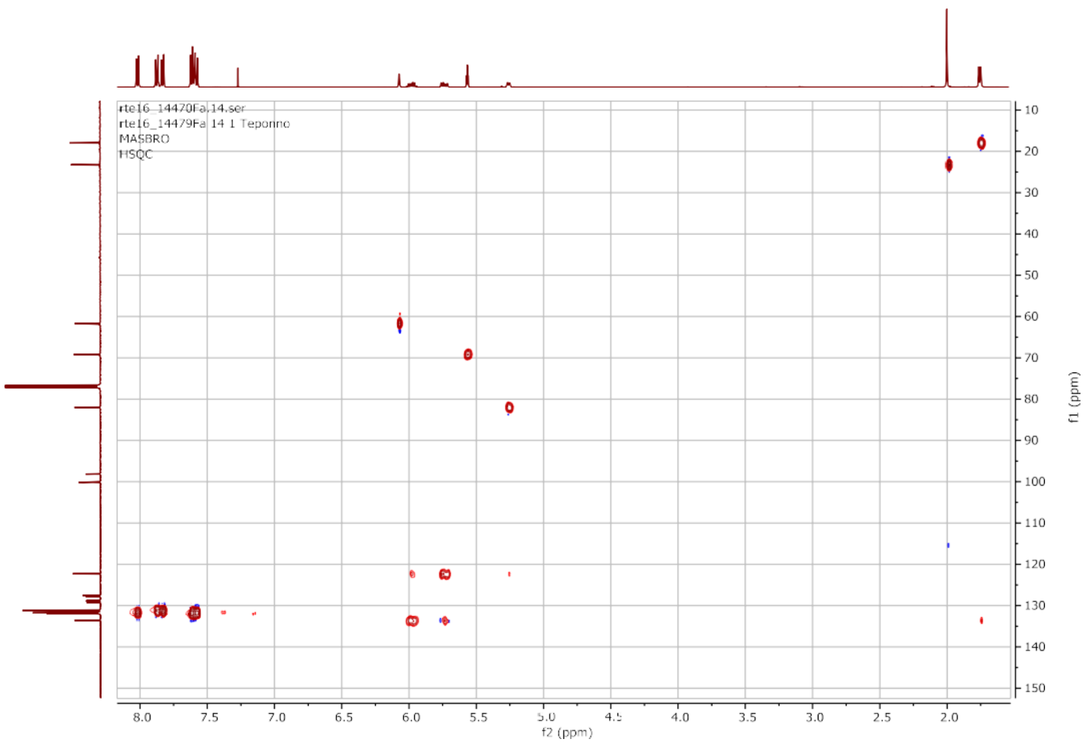


**Figure S31**: HSQC spectrum of compound **5**.

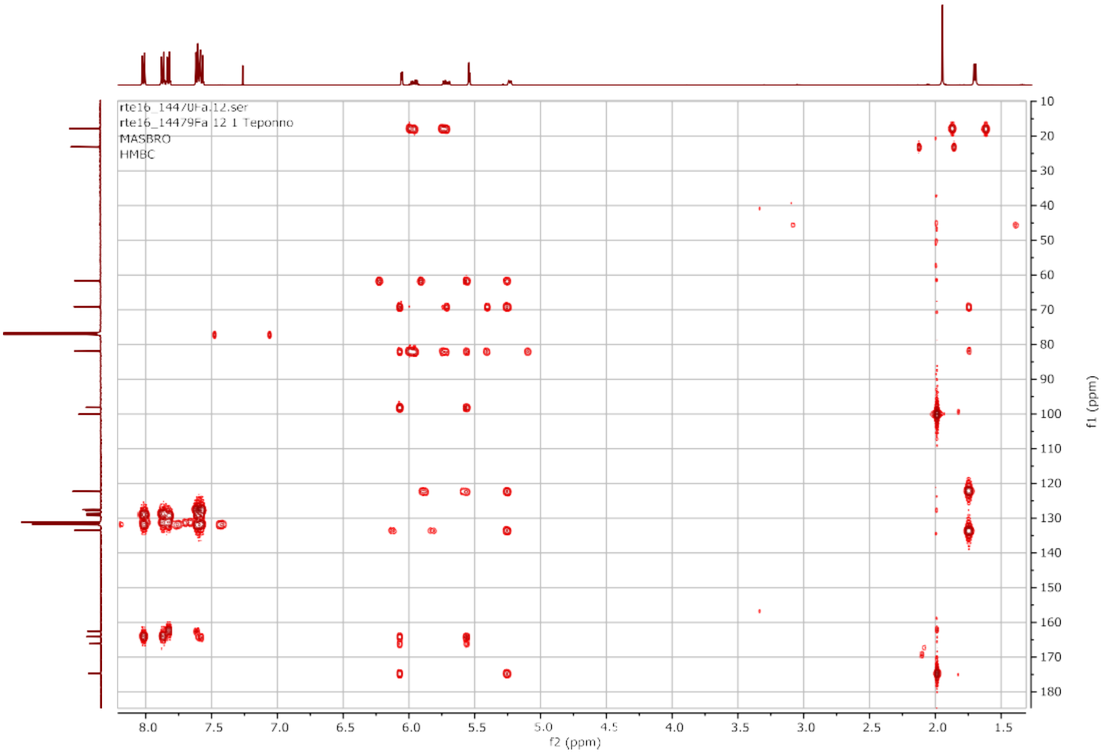


**Figure S32**: HMBC spectrum of compound **5**.

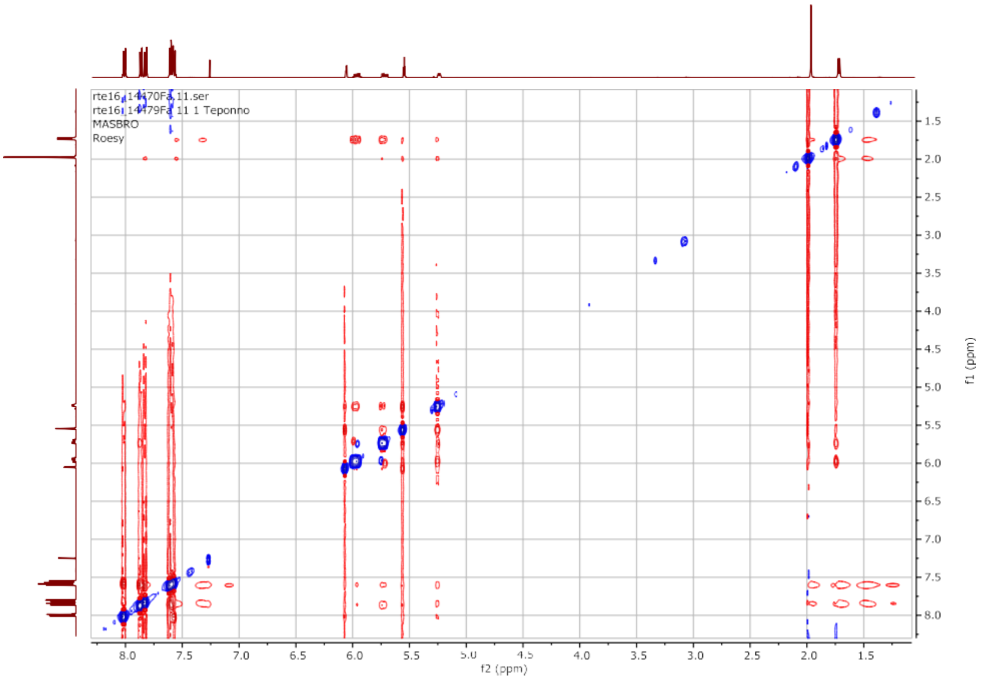


**Figure S33**: ROESY spectrum of compound **5**.

## Spectrum Peak Pick Report

20.04.2018 13:09:12

Data Set: MAS-BRO-F6-0.0375 - RawData

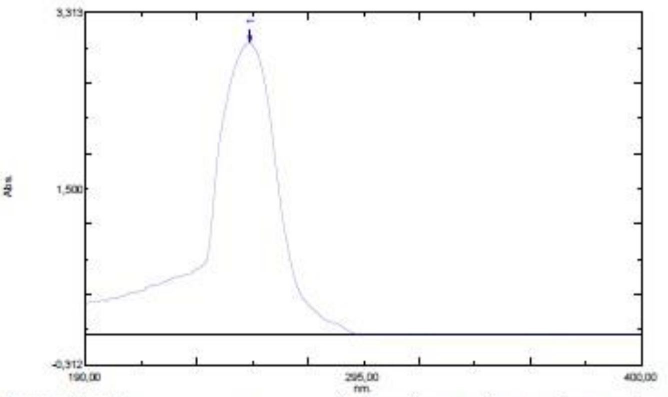


| No. | P/V | Wavelength | Abs. | Description |
|---|---|---|---|---|
| 1 | | 252,00 | 3,011 | |
| 2 | | 315,00 | -0,010 | |

| | |
|---|---|
| **Measurement Properties** | |
| Wavelength Range (nm.): | 190,00 to 400,00 |
| Scan Speed: | Medium |
| Sampling Interval: | 1,0 |
| Auto Sampling Interval: | Disabled |
| Scan Mode: | Single |
| **Instrument Properties** | |
| Instrument Type: | UV-2400PC Series |
| Measuring Mode: | Absorbance |
| Slit Width: | 2,0 nm |
| Light Source Change Wavelength: | 360,0 nm |
| S/R Exchange: | Normal |
| **Attachment Properties** | |
| Attachment: | None |
| **Sample Preparation Properties** | |
| Weight: | 0.0375 |
| Volume: | 1mL |
| Dilution: | |
| Path Length: | 1 cm |
| Additional Information: | MAS-BRO-F6-0.075 |

**Figure S34**: UV spectrum of compound **5**.

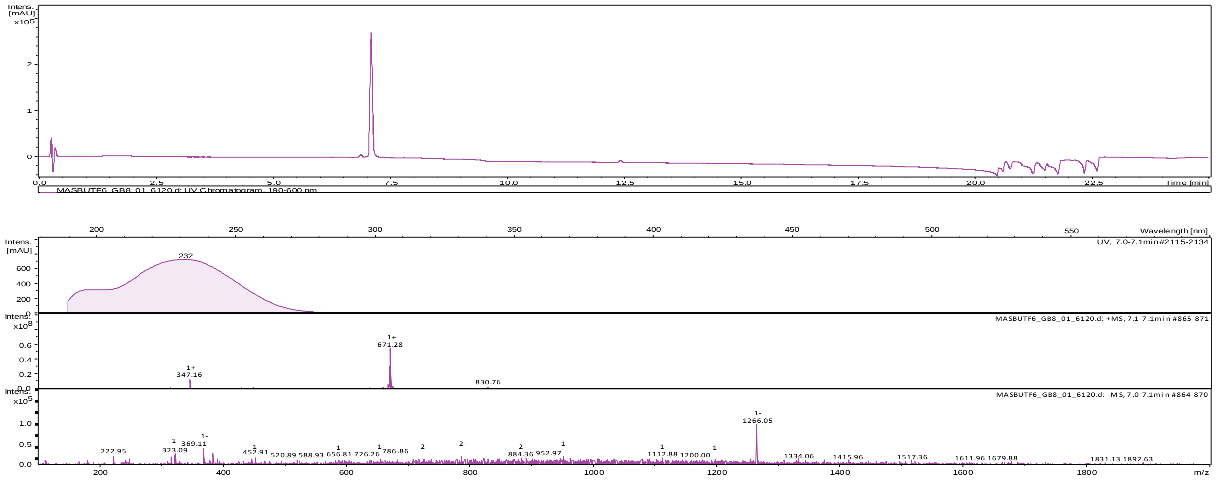


**Figure S35:** LC-UV-ESIMS spectra of compound **6**

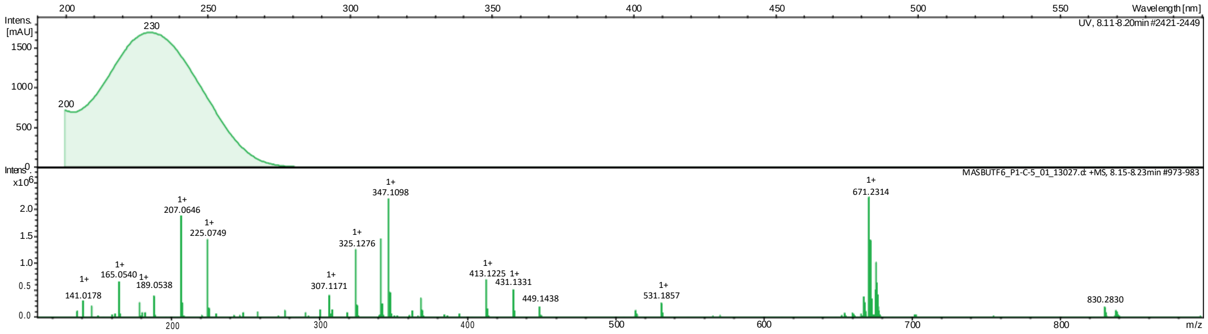


**Figure S36**: UV-HRESIMS spectra of compound **6**.

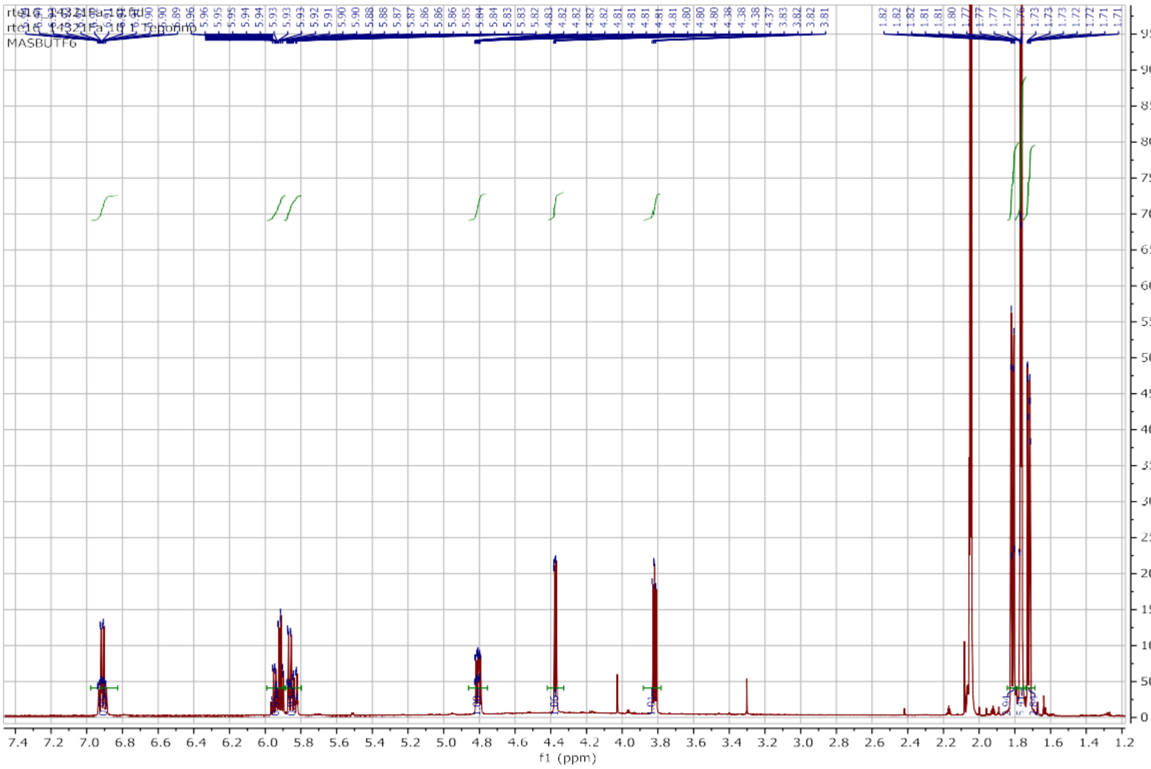


**Figure S37**: $^{1}$H NMR spectrum of compound **6** in acetone-$d_6$ at 500 MHz.

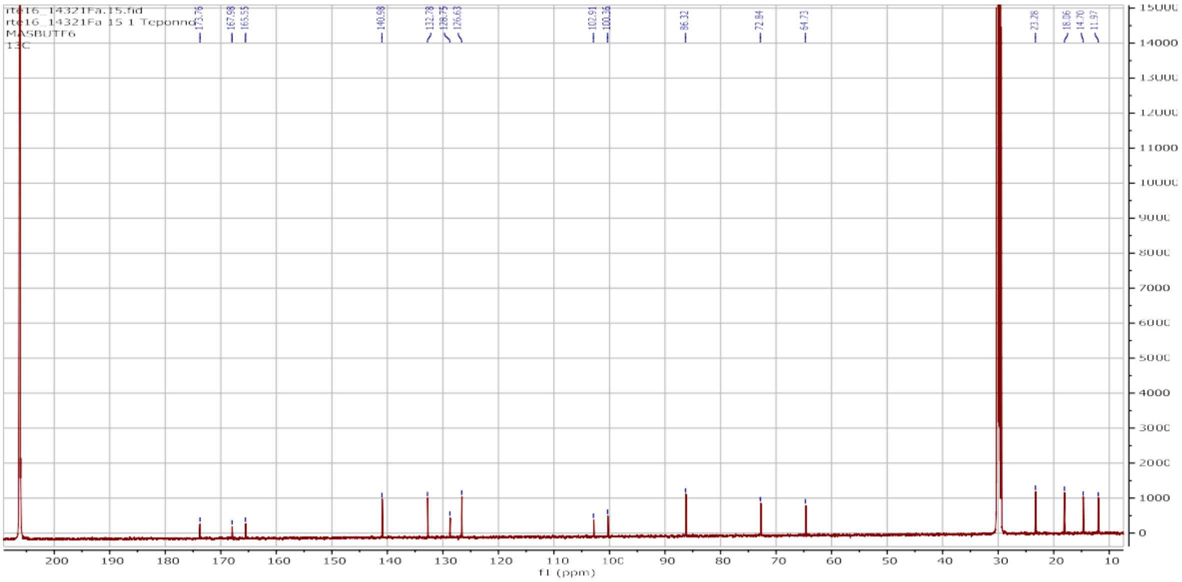


**Figure S38**: $^{13}$C NMR spectrum of compound **6** in acetone-$d_6$ at 125 MHz.

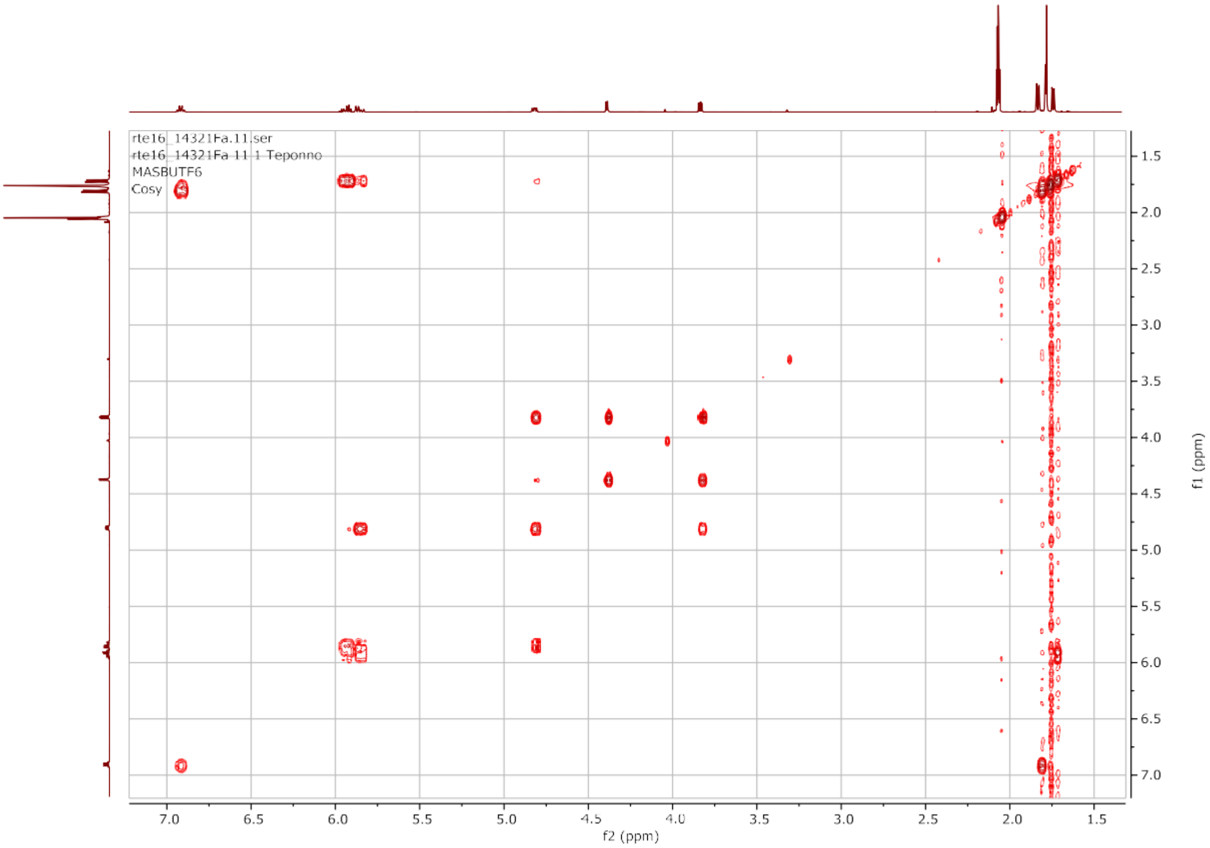


**Figure S39**: $^1$H-$^1$H spectrum of compound **6** in acetone-$d_6$.

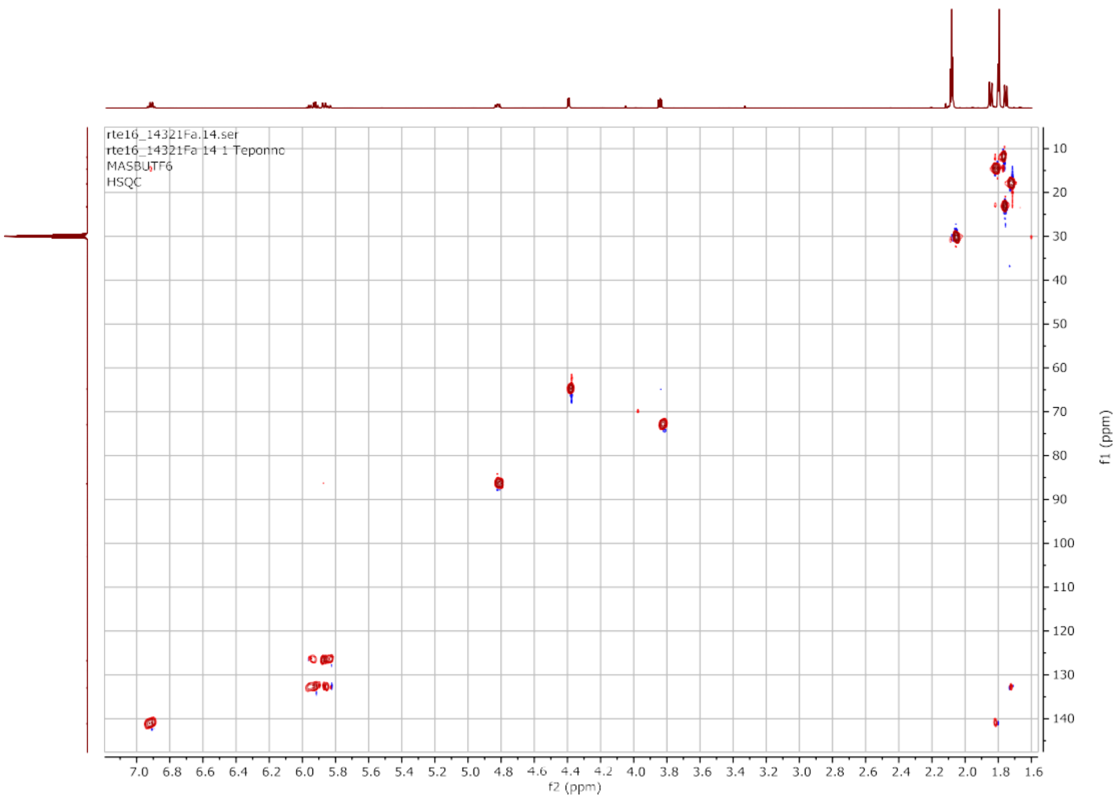


**Figure S40**: HSQC spectrum of compound **6** in acetone-$d_6$.

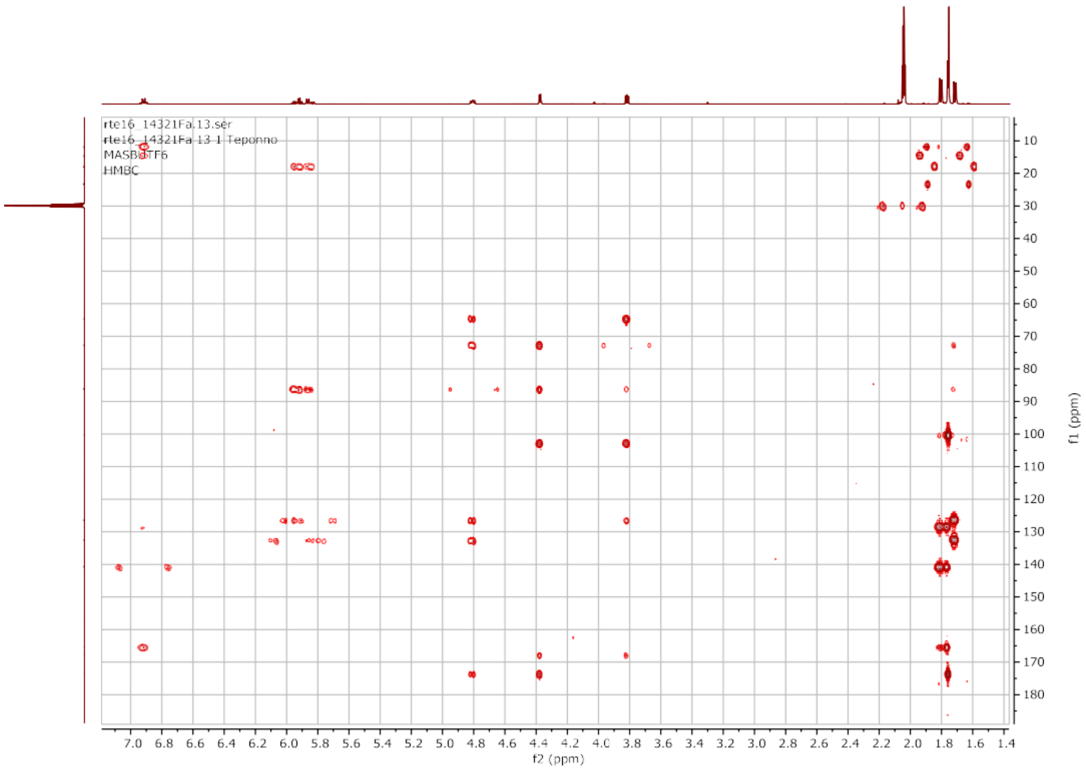


**Figure S41**: HMBC spectrum of compound **6** in acetone-$d_6$.

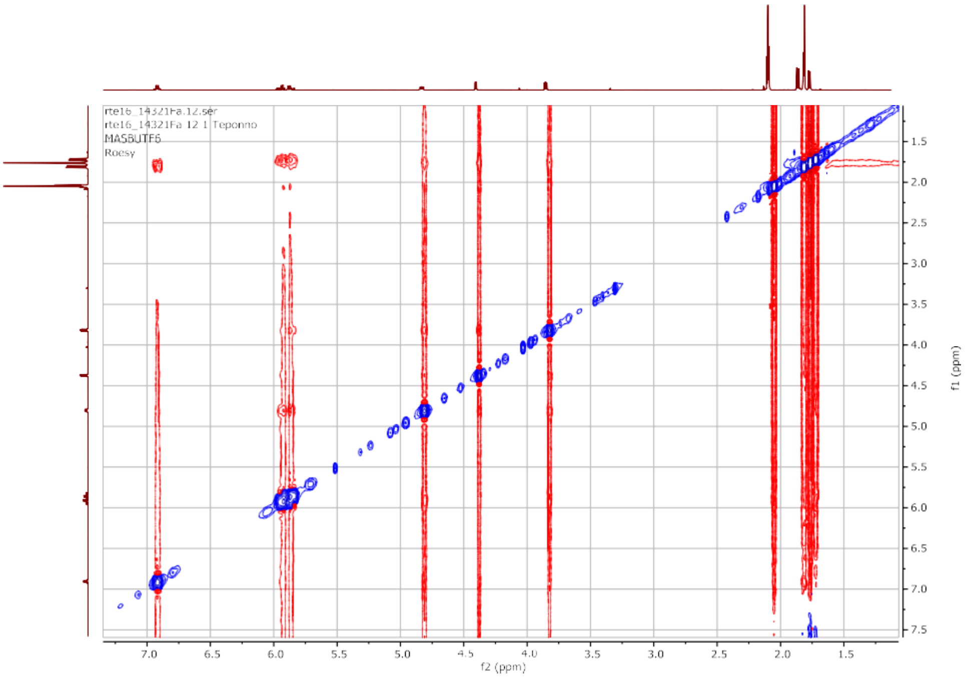


**Figure S42**: ROESY spectrum of compound **6** in acetone-$d_6$.

## Spectrum Peak Pick Report

20.04.2018 12:54:06

Data Set: MAS-BUT-F6-0.15 - RawData

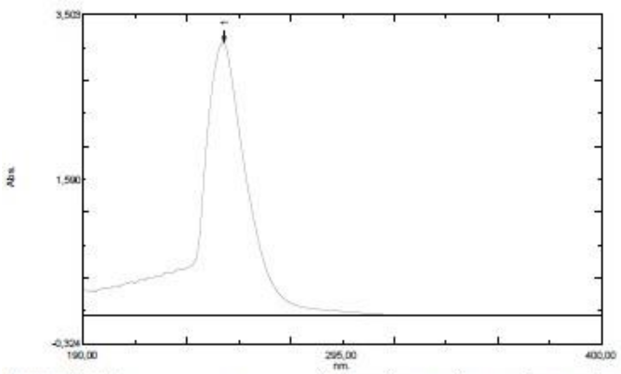


| No. | P/V | Wavelength | Abs. | Description |
|---|---|---|---|---|
| 1 | | 247,00 | 3,184 | |

Measurement Properties
Wavelength Range (nm.): 190,00 to 400,00
Scan Speed: Medium
Sampling Interval: 1,0
Auto Sampling Interval: Disabled
Scan Mode: Single

Instrument Properties
Instrument Type: UV-2400PC Series
Measuring Mode: Absorbance
Slit Width: 2,0 nm
Light Source Change Wavelength: 360,0 nm
S/R Exchange: Normal

Attachment Properties
Attachment: None

Sample Preparation Properties
Weight: 0.15
Volume: 1mL
Dilution:
Path Length: 1 cm
Additional Information: MAS-BUT-F6

**Figure S43**: UV spectrum of compound **6**.

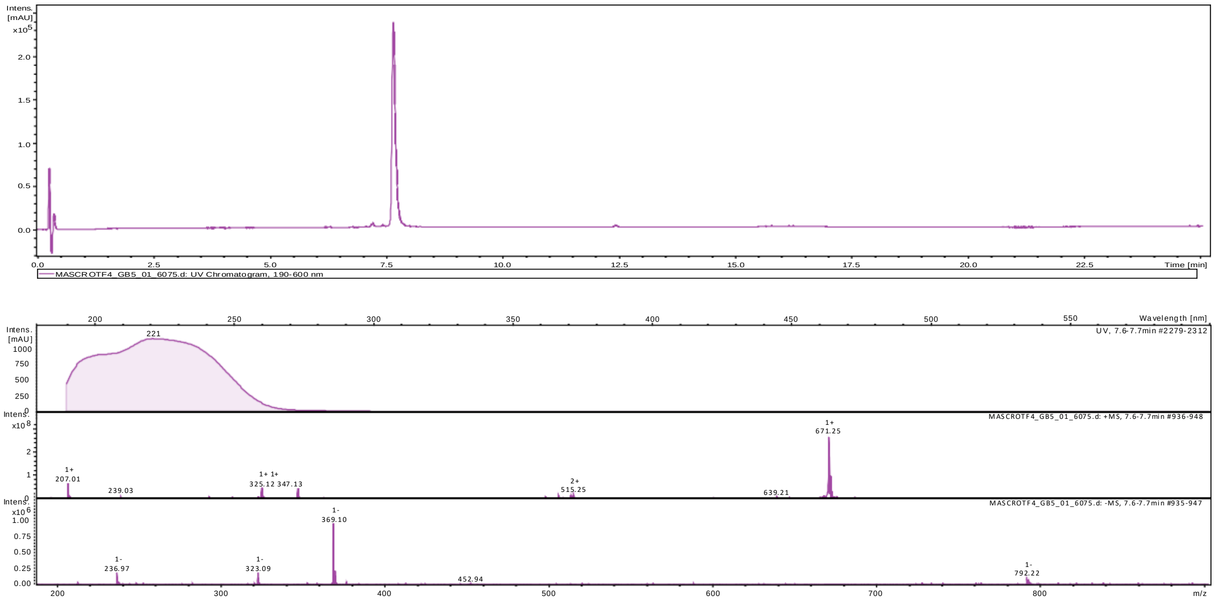


**Figure S44**: LC-UV ESIMS spectra of compound **7**

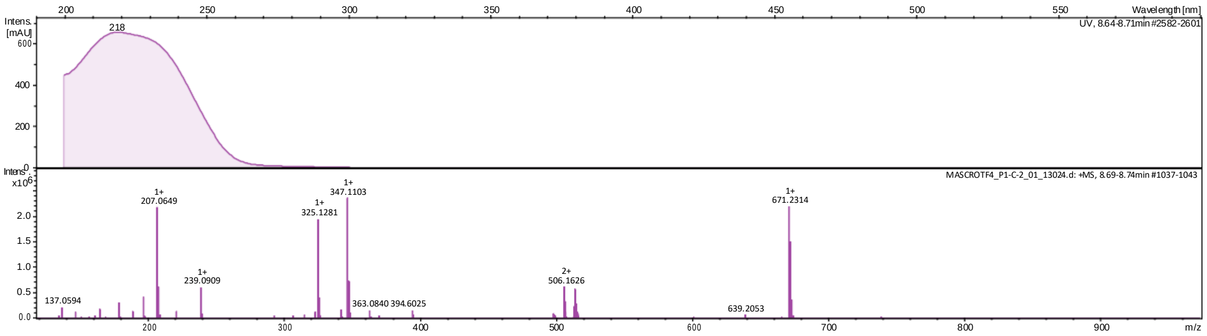


**Figure S45**: UV-HRESIMS spectrum of compound **7**.

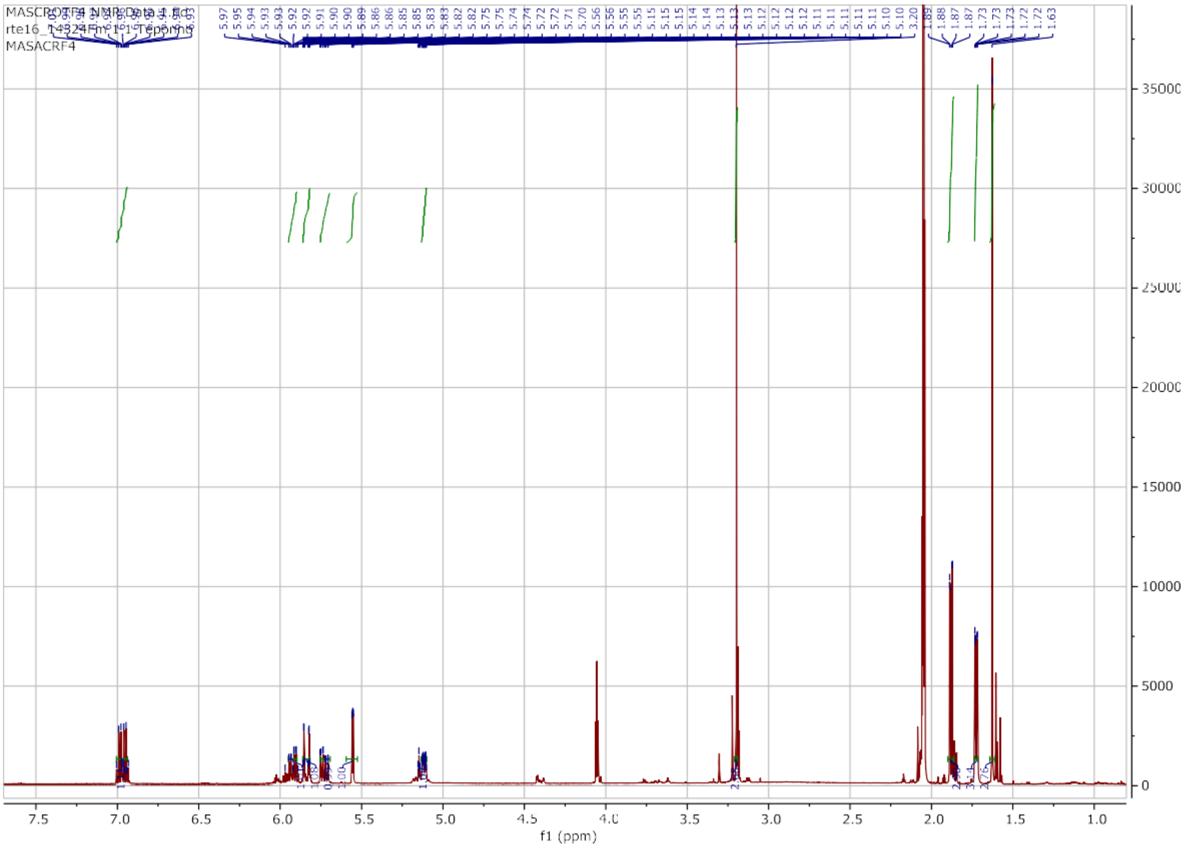

**Figure S46**: $^{1}$H NMR spectrum of compound **7** in acetone-$d_6$ at 500 MHz.

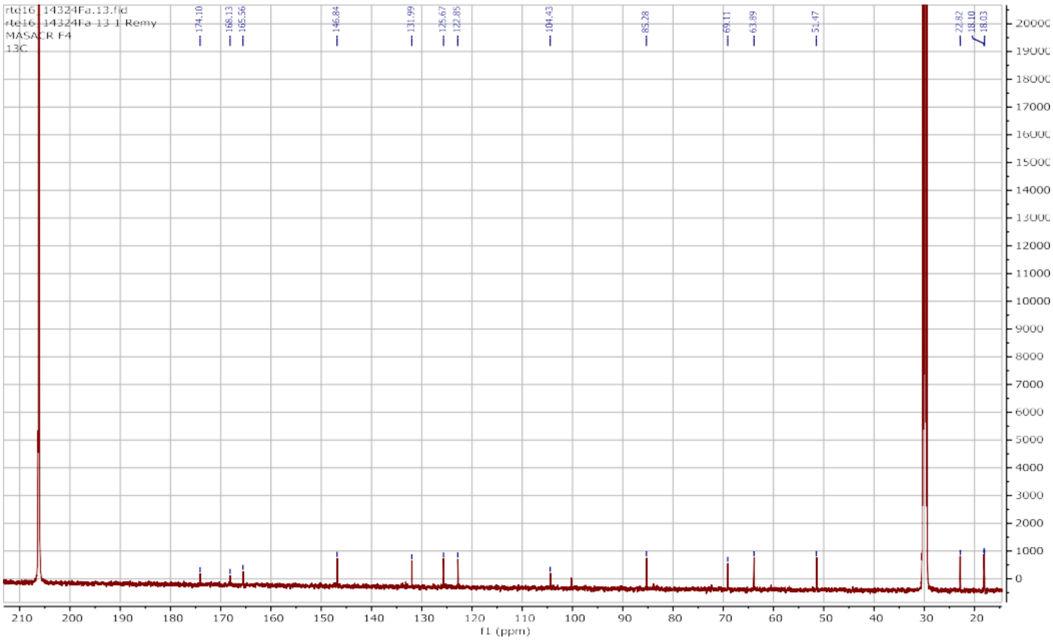

**Figure S47**: $^{13}$C NMR spectrum of compound **7** in acetone-$d_6$ at 125 MHz.

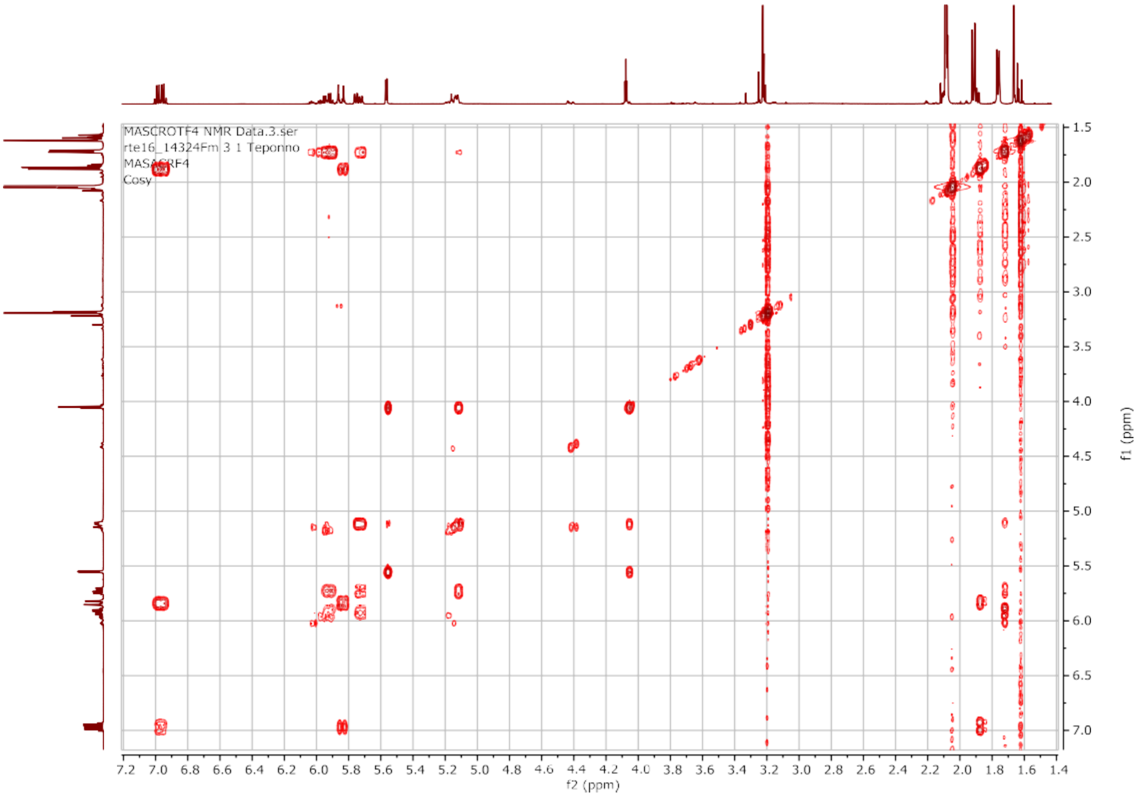


**Figure S48**: $^{1}H$-$^{1}H$ COSY spectrum of compound **7** in acetone-$d_6$.

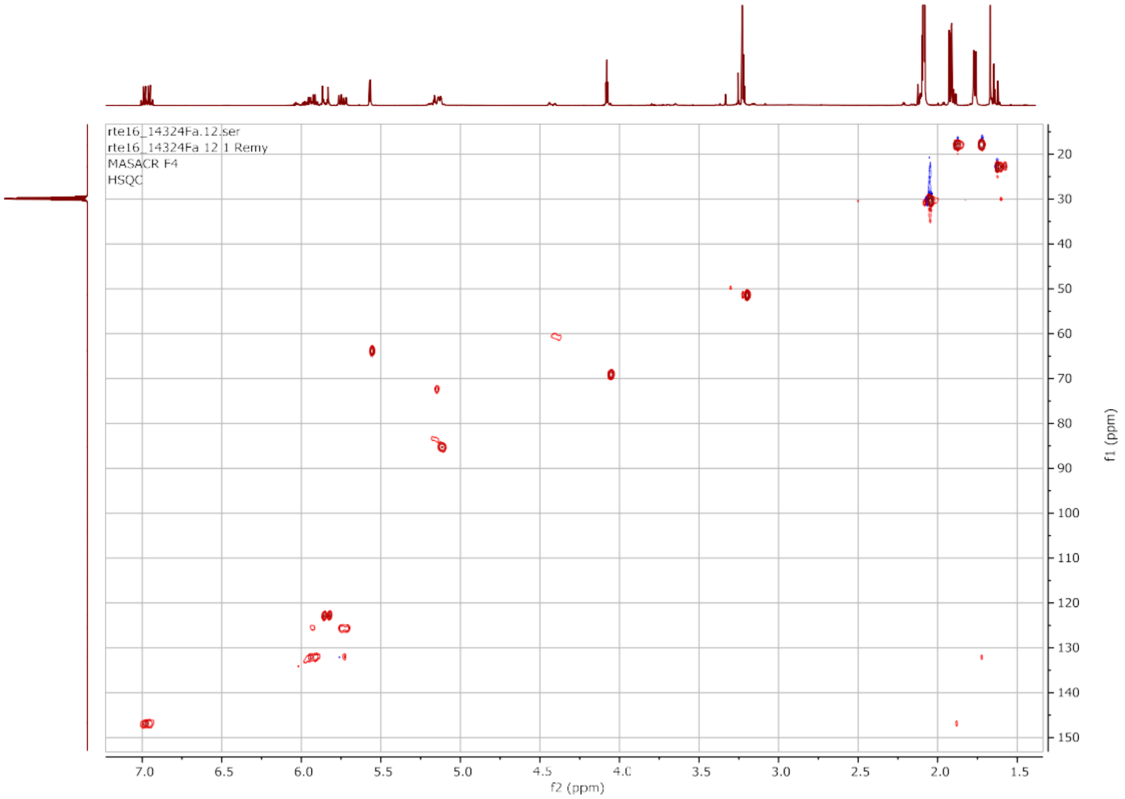


**Figure S49**: HSQC spectrum of compound **7** in acetone-$d_6$.

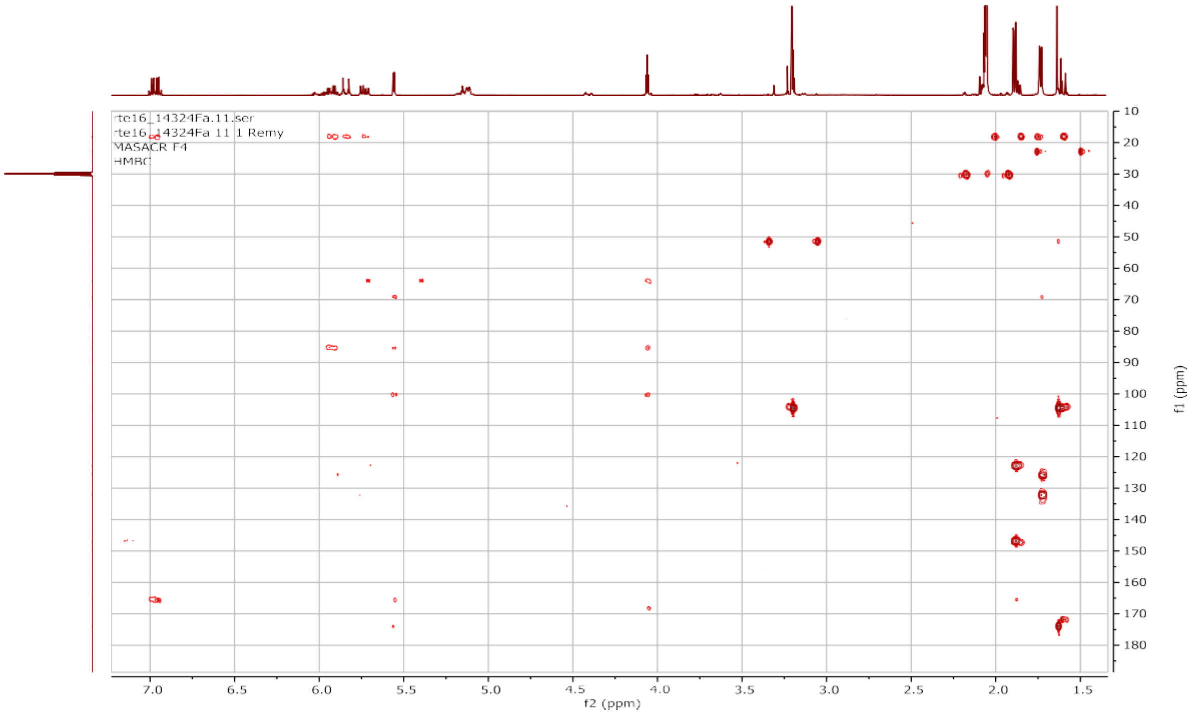


**Figure S50**: HMBC spectrum of compound **7** in acetone-$d_6$.

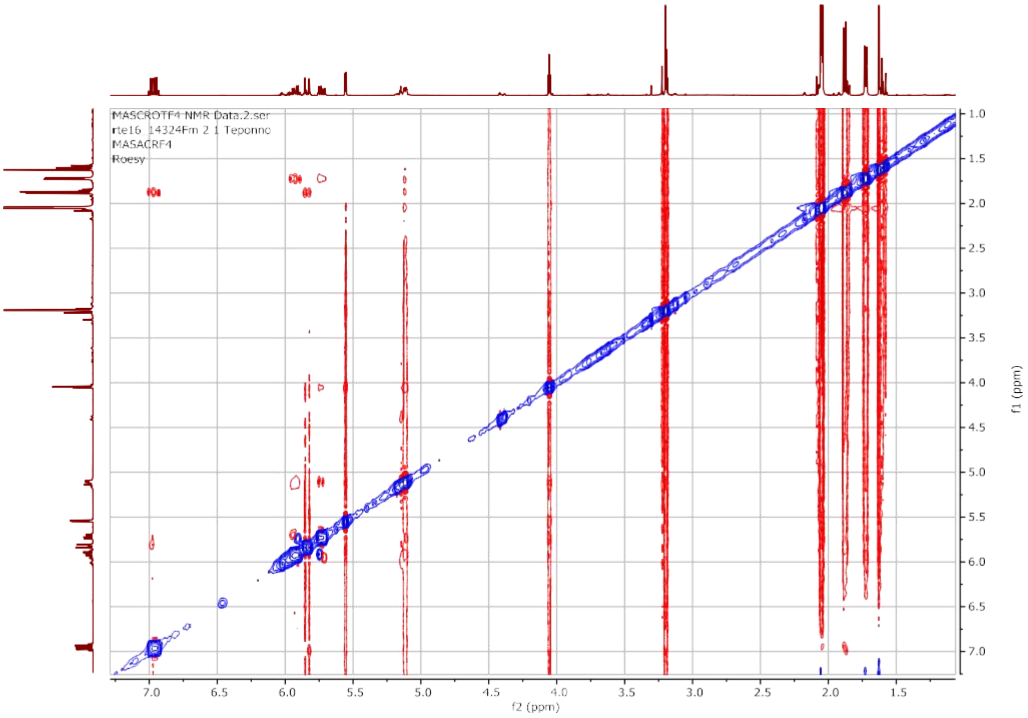


**Figure S51**: ROESY spectrum of compound **7** in acetone-$d_6$.

## Spectrum Peak Pick Report

20.04.2018 13:40:51

Data Set: MAS-CROT-F4-0.075 - RawData

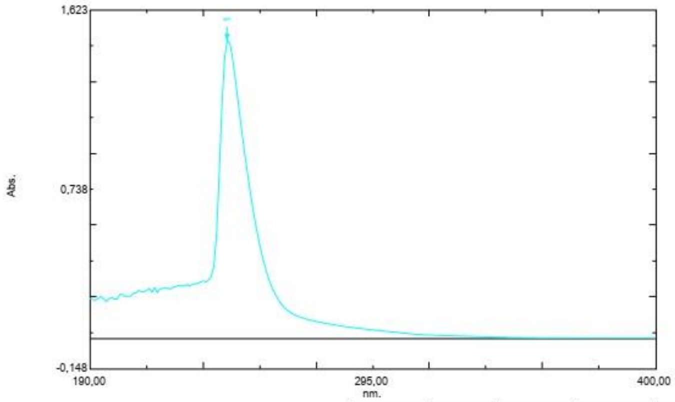


| Measurement Properties | |
|---|---|
| Wavelength Range (nm.): | 190,00 to 400,00 |
| Scan Speed: | Medium |
| Sampling Interval: | 1,0 |
| Auto Sampling Interval: | Disabled |
| Scan Mode: | Single |

| No. | P/V | Wavelength | Abs. | Description |
|---|---|---|---|---|
| 1 | | 241,00 | 1,476 | |

| Instrument Properties | |
|---|---|
| Instrument Type: | UV-2400PC Series |
| Measuring Mode: | Absorbance |
| Slit Width: | 2,0 nm |
| Light Source Change Wavelength: | 360,0 nm |
| S/R Exchange: | Normal |

| Attachment Properties | |
|---|---|
| Attachment: | None |

| Sample Preparation Properties | |
|---|---|
| Weight: | 0.01875 |
| Volume: | 1mL |
| Dilution: | |
| Path Length: | 1 cm |
| Additional Information: | MAS-CROT-F4-0.075 |

**Figure S52**: UV spectrum of compound **7**.

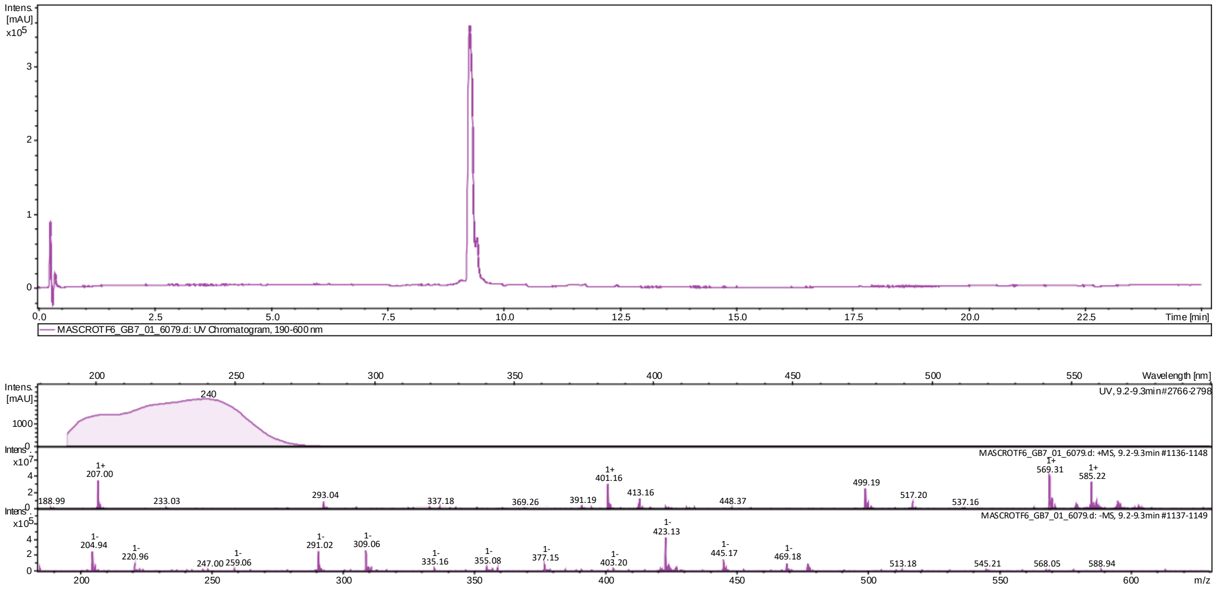


**Figure S53**: LC-UV-ESIMS spectrum of compound **8**.

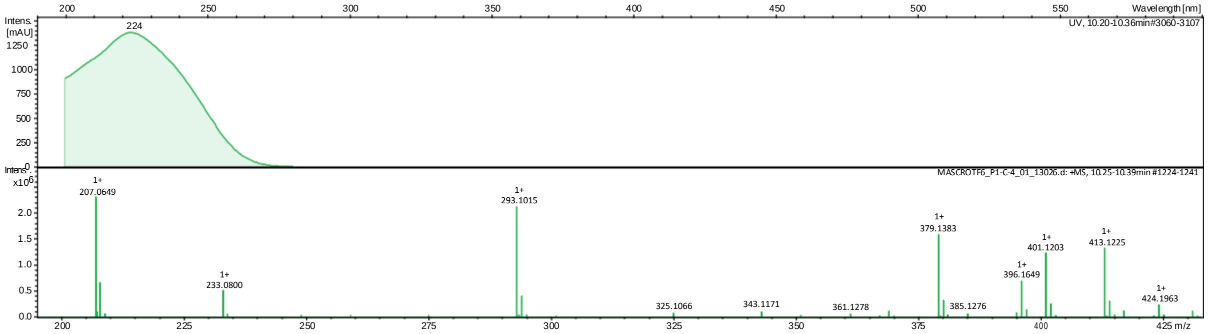


**Figure S54**: UV-HRESIMS spectrum of compound **8**.

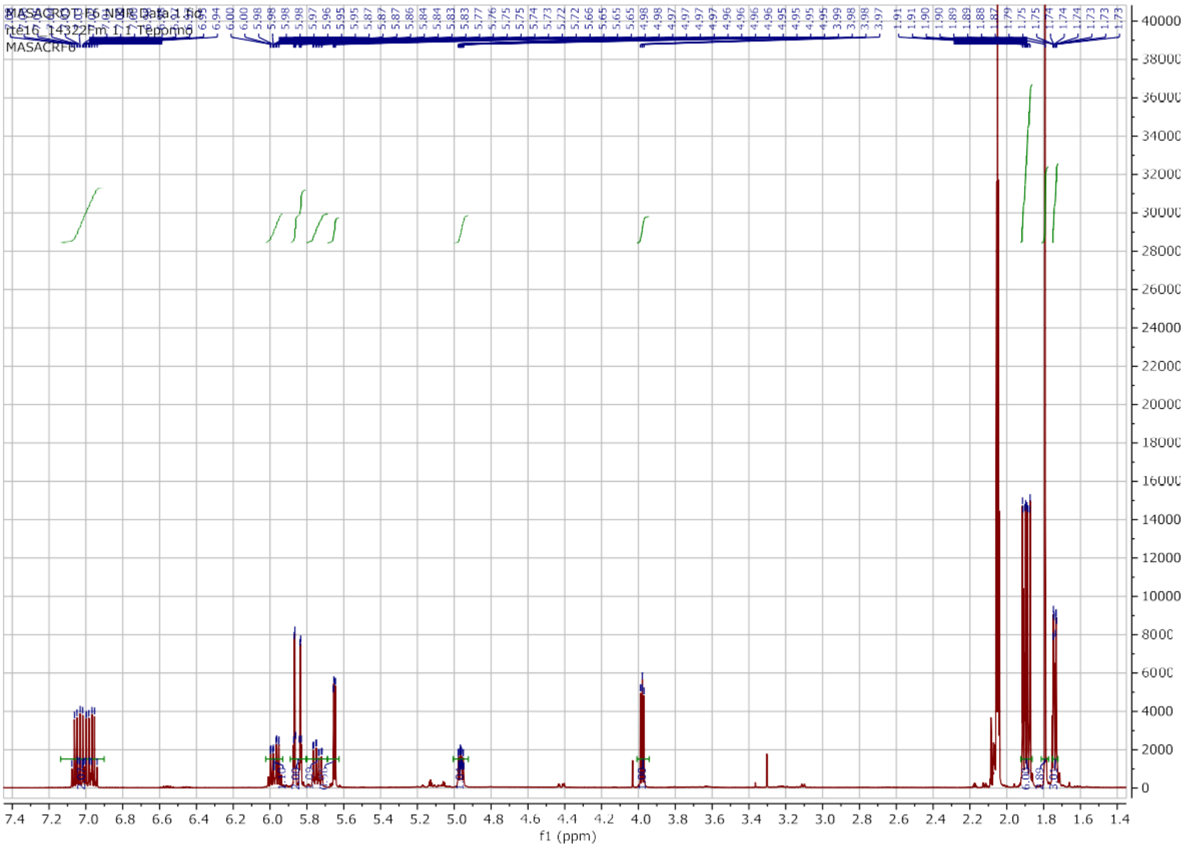


**Figure S55**: $^{1}$H NMR spectrum of compound **8** in acetone-$d_6$ at 500 MHz.

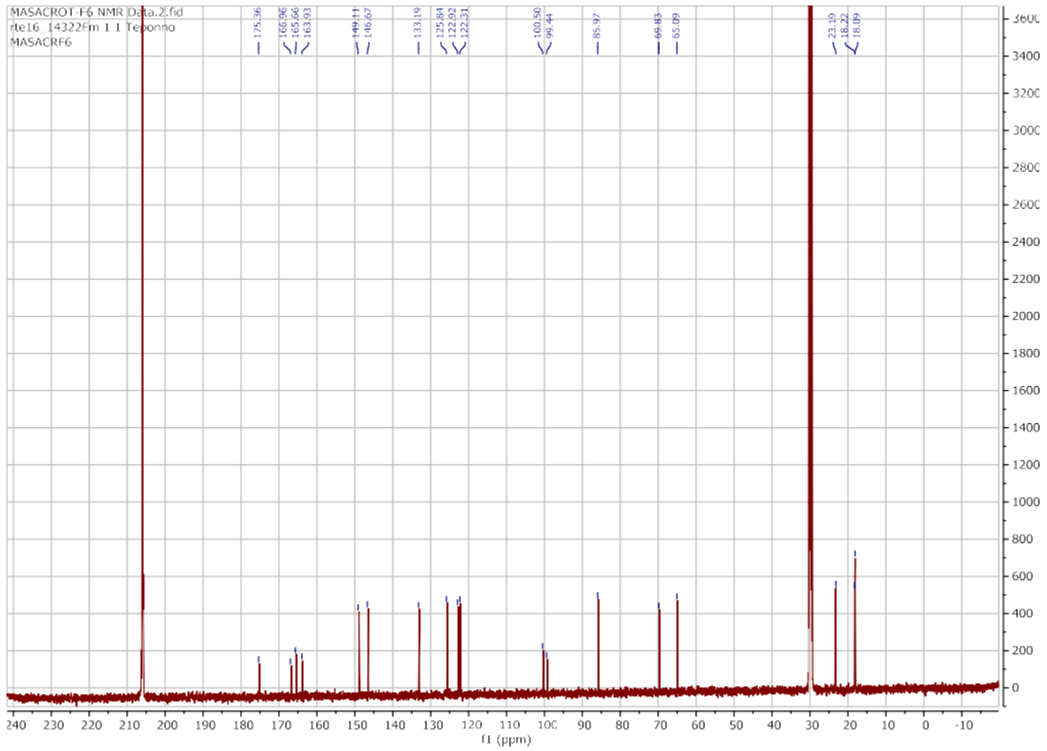

**Figure S56**: $^{13}C$ NMR spectrum of compound **8** in acetone-$d_6$ at 125 MHz.

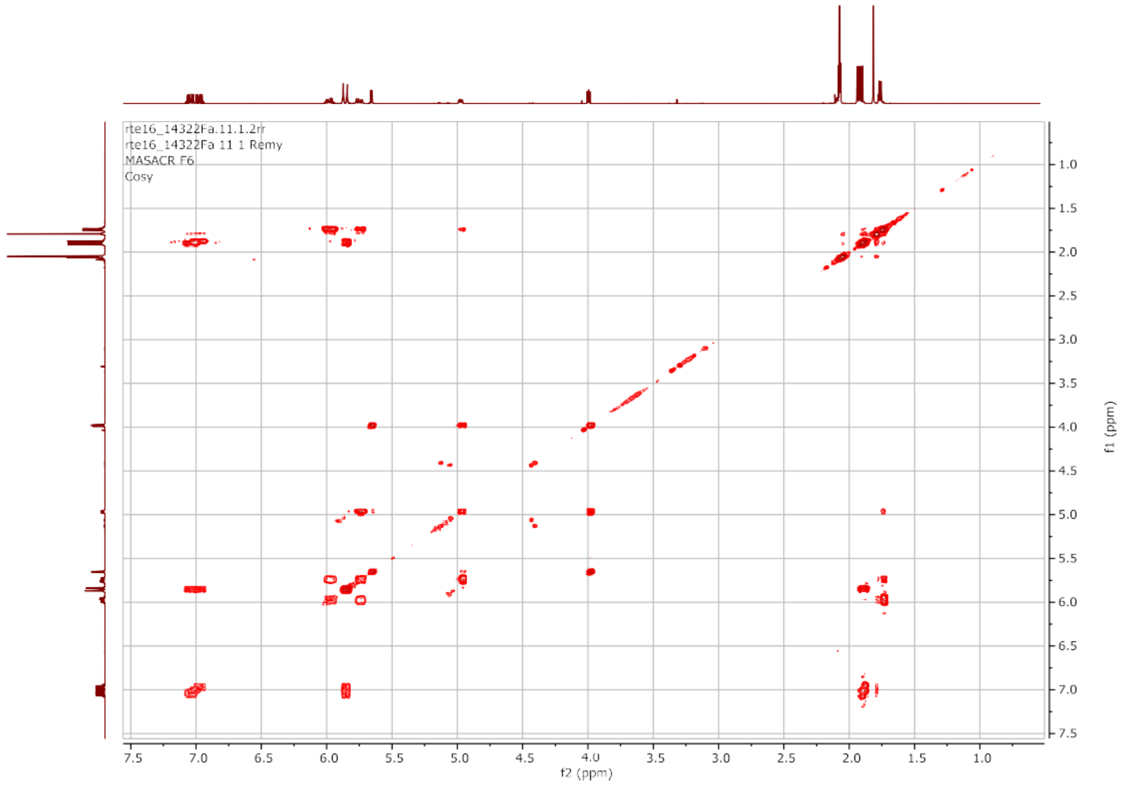

**Figure S57**: $^1H$-$^1H$ COSY spectrum of compound **8** in acetone-$d_6$.

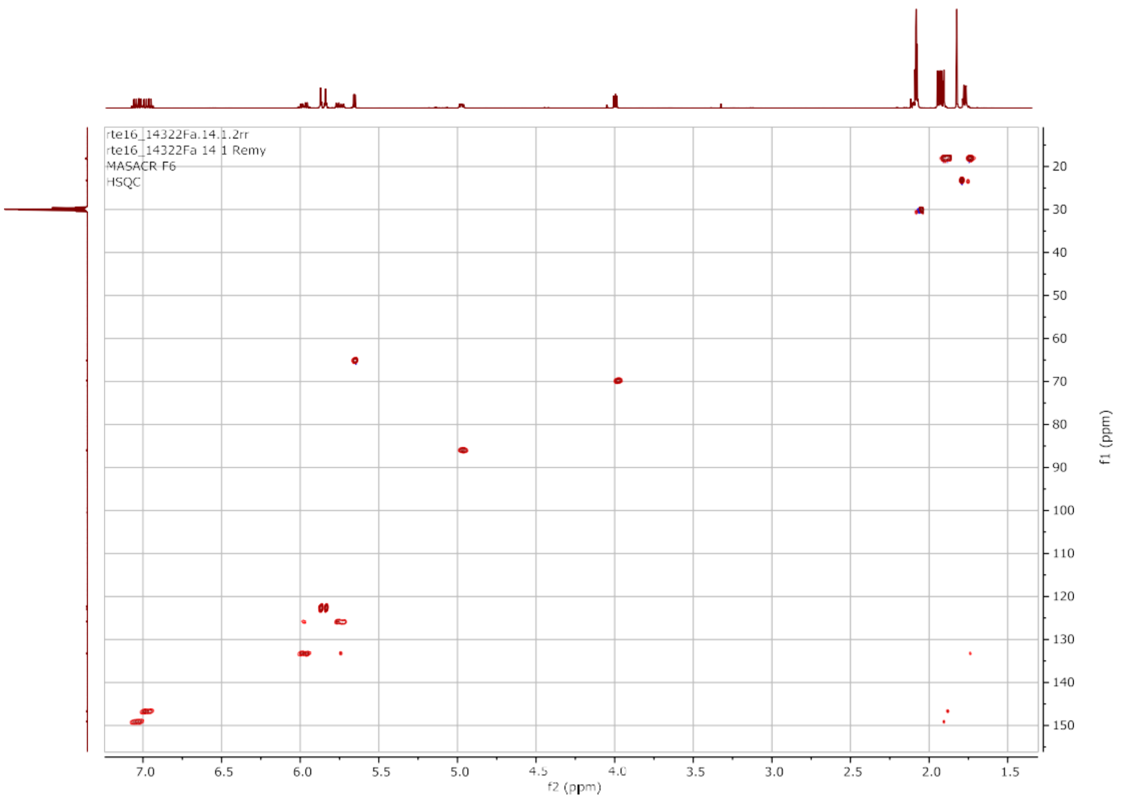


**Figure S58**: HSQC spectrum of compound **8** in acetone-$d_6$.

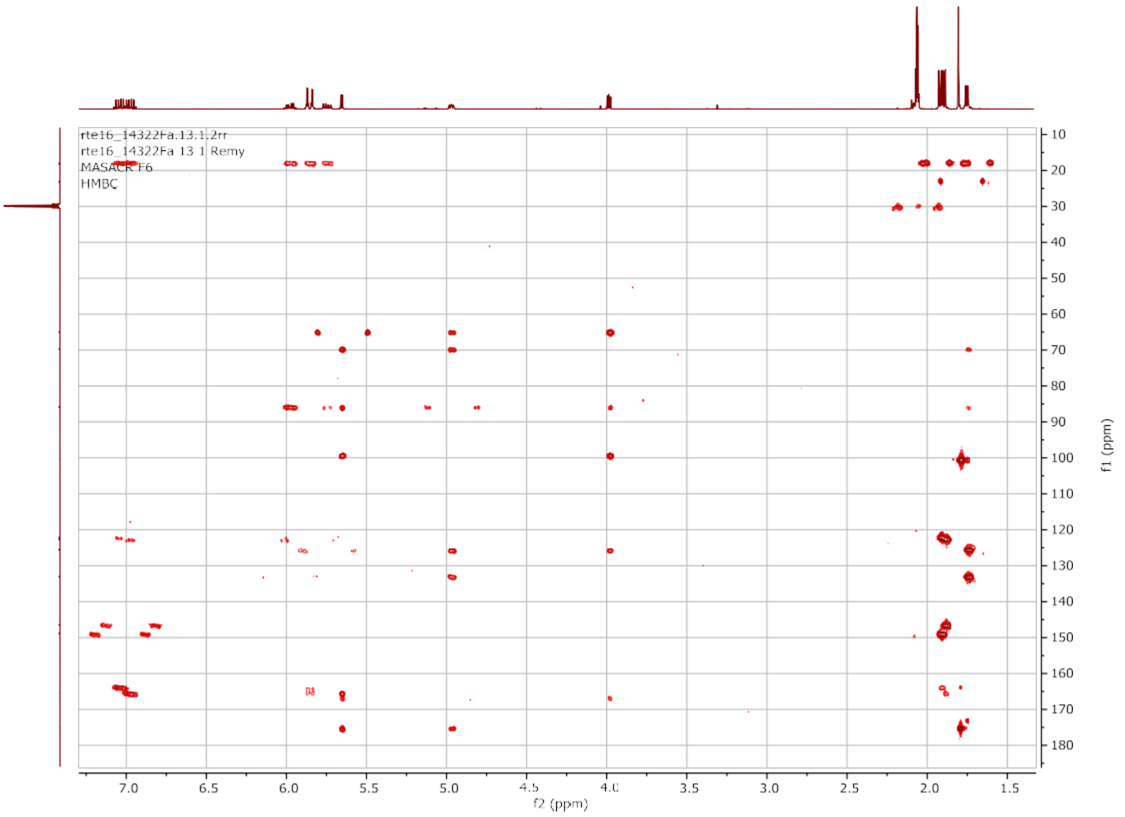


**Figure S59**: HMBC spectrum of compound **8** in acetone-$d_6$.

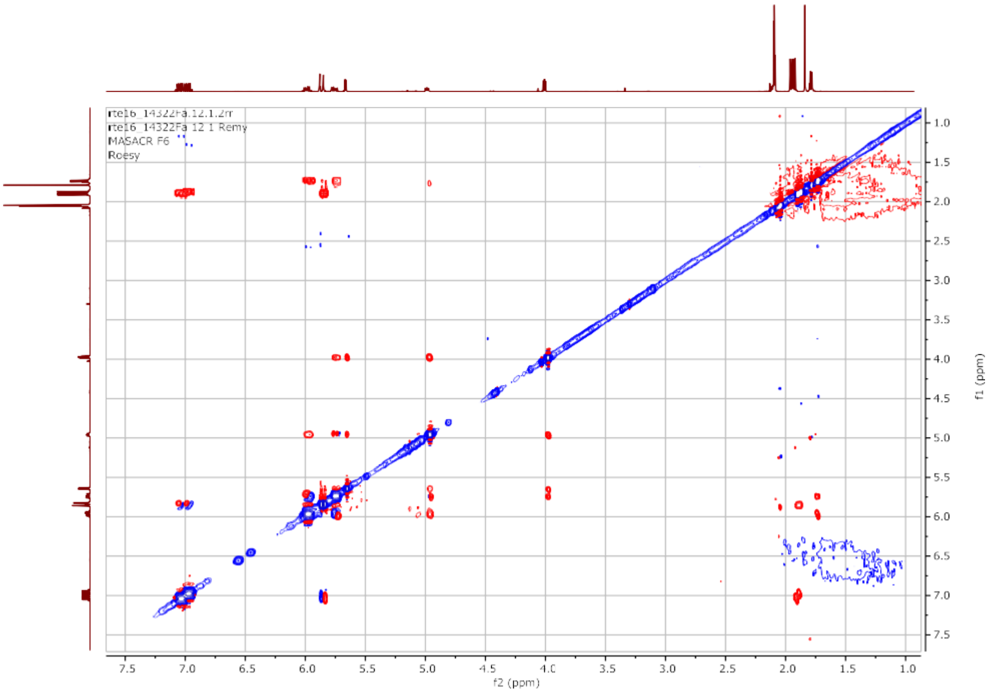


**Figure S60**: ROESY spectrum of compound **8** in acetone-$d_6$.

## Spectrum Peak Pick Report

20.04.2018 12:59:29

Data Set: MAS-CROT-F6-0.075 - RawData

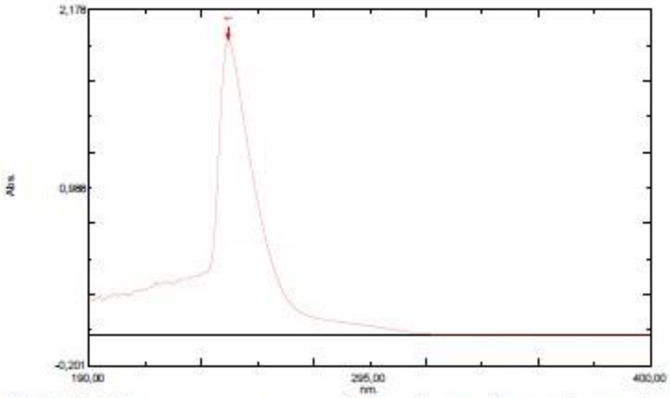


| No. | P/V | Wavelength | Abs. | Description |
|---|---|---|---|---|
| 1 |  | 242,00 | 1,979 |  |

| | |
|---|---|
| **Measurement Properties** | |
| Wavelength Range (nm.): | 190,00 to 400,00 |
| Scan Speed: | Medium |
| Sampling Interval: | 1,0 |
| Auto Sampling Interval: | Disabled |
| Scan Mode: | Single |
| **Instrument Properties** | |
| Instrument Type: | UV-2400PC Series |
| Measuring Mode: | Absorbance |
| Slit Width: | 2,0 nm |
| Light Source Change Wavelength: | 360,0 nm |
| S/R Exchange: | Normal |
| **Attachment Properties** | |
| Attachment: | None |
| **Sample Preparation Properties** | |
| Weight: | 0.075 |
| Volume: | 1mL |
| Dilution: | |
| Path Length: | 1 cm |
| Additional Information: | MAS-CROT-F6-0.075 |

**Figure S61**: UV spectrum of compound **8**.

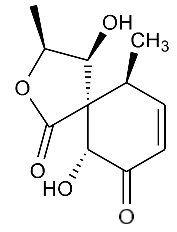

Rosigenin

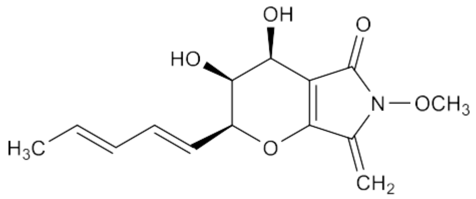

Curvupallide A

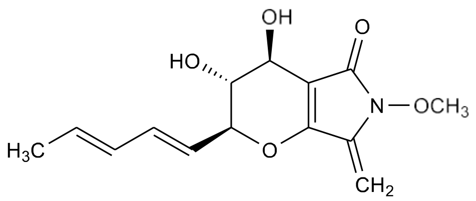

Curvupallide B

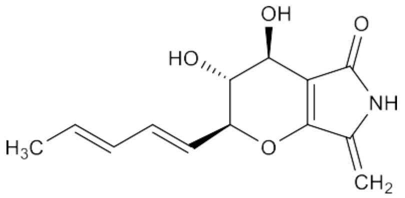

Curvupallide C

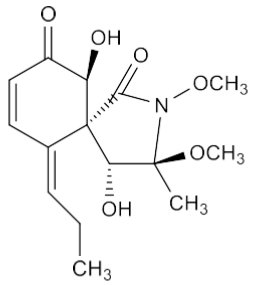

Spirostaphylotrichin X

**Figure S62**: Chemical structures of rosigenin, curvupallides A–C, and spirostaphylotrichin X.
